# The dual HDAC/PI3K inhibitor CUDC-907 inhibits the growth and proliferation of MYC-driven Group 3 medulloblastoma

**DOI:** 10.1038/s41420-025-02470-4

**Published:** 2025-04-14

**Authors:** Pan Gou, Chencheng Fang, Man Xu, Dandan Zhang, Xuanxuan Wu, Li Zhang, Xiao Li, Man Li, Lu Gan, Jinjin Luo, Hongjuan Cui, Ping Liang

**Affiliations:** 1 Department of Neurosurgery Children’s Hospital of Chongqing Medical University, National Clinical Research Center for Child Health and Disorders, Ministry of Education Key Laboratory of Child Development and Disorders, Chongqing Key Laboratory of Child Neurodevelopment and Cognitive Disorders, Chongqing, China; 2 Jinfeng Laboratory, Chongqing, China; 3 Medical Research Institute, State Key Laboratory of Resource Insects, Southwest University, Chongqing, China

**Keywords:** CNS cancer, Target validation, Gene expression, Preclinical research, Drug development

## Abstract

Metastatic Group 3 medulloblastoma (G3 MB) have been shown in several studies to be very high risk, particularly those harboring MYC amplification. More effective therapies are especially important for these patients. CUDC-907, a novel dual inhibitor targeting the MYC upstream pathway (HDAC/PI3K), shows significant antitumor efficacy across multiple cancer types. However, the antitumor effects and underlying mechanisms of CUDC-907 in MB, particularly in very high-risk MB, remain unexplored. In this study, we showed that MYC amplified G3 MB cells, patient-derived organoids and xenograft models were sensitive to CUDC-907. CUDC‐907 inhibited MYC expression through the HDAC and PI3K pathways, and then induced G0/G1 phase arrest through the MYC-P21/P27-CDKs/cyclins axis. Furthermore, when CUDC-907 was combined with chemotherapeutic drug cisplatin, G0/G1 phase blocking effect was further enhanced. CUDC-907 in combination with radiotherapy (RT) inhibited DNA damage repair and increased DNA damage. These findings indicate that CUDC-907, either as a monotherapy or in combination with chemoradiotherapy, represents a promising therapeutic strategy for MYC amplified G3 MB, potentially influencing future clinical trials targeting this patient population.

## Introduction

As one of the most common malignant brain tumors in children, the World Health Organization classified medulloblastoma (MB) into four subtypes on the basis of genomics and DNA methylation: WNT, SHH, and non-WNT/non-SHH (Group 3 and Group 4) [1, 2]. The 2015 Heidelberg Conference further redefined the risk classification on the basis of the molecular type of methylation. Multiple studies, including the MAGIC consortium, HIT2000, and the UK research cohort, have demonstrated that metastatic Group 3 medulloblastoma patients exhibit a poor prognosis, particularly those with MYC amplification. Even if children receive comprehensive treatment, such as maximal safe resection and high-dose chemoradiotherapy, the five-year survival rate is still less than 50% [3–5]. Moreover, high-intensity chemotherapy and radiotherapy (RT) undoubtedly cause long-term sequelae such as neurological dysfunction, endocrine disorders, and cognitive decline [6, 7]. Therefore, there is an urgent need for further research to identify specific therapeutic agents effective against this group.

MYC is one of the most common dysregulated oncogenes in human cancers, which coordinates extensive transcriptional changes and plays a central role in tumor development and maintenance [8]. The overexpression of MYC has been shown to be associated with poor prognosis in some solid tumors, such as breast cancer, ovarian cancer, prostate cancer and lung cancer [9–12]. MYC is also amplified or overexpressed among G3 tumors which is the most aggressive forms of MB. These patients are more likely to exhibit drug resistance, early metastasis, and recurrence after treatment [13, 14]. Unfortunately, despite MYC being one of the most extensively implicated oncogenes, it has been demonstrated that directly targeting MYC as a regulator of catalytic transcriptional activity remains a significant challenge [15–17]. Increasing evidence suggests that targeting upstream regulatory factors such as histone deacetylase (HDAC), phosphoinositol 3-kinase (PI3K), polo-like kinase 1 (PLK1) and BET bromodomain can lower MYC protein levels and inhibit MYC-driven cancer growth [18–21].

At present, CUDC-907, an orally bio-available small molecule dual HDAC (class I, II, and IV, including HDAC1, 2, 3, 6, 10, and 11) and PI3K (α, β, and δ) inhibitor, has been reported to exhibit broad-spectrum antitumor activity [22]. In addition, phase I and II clinical trials for the treatment of multiple myeloma, lymphoma, and advanced/recurrent solid tumors in adults and children are underway (www.clinicalTrials.gov). According to existing studies on CUDC-907, downregulation of MYC protein is an early event induced by CUDC-907 therapy that restricts MYC-driven cancer growth [23, 24]. Our preclinical results demonstrate the antitumor effects of CUDC-907 in G3 MB and further explored the mechanism how CUDC-907 affects MB cell growth and tumorigenicity by suppressing MYC. Moreover, we found that CUDC-907 sensitized MB cells to both chemotherapy and RT in vivo and in vitro. These findings may provide evidence for the clinical translation of CUDC-907 in MYC amplified, metastatic G3 MB patients.

## Results

### MYC is highly expressed in G3 MB and significantly correlated with prognosis

**Fig. 1:**
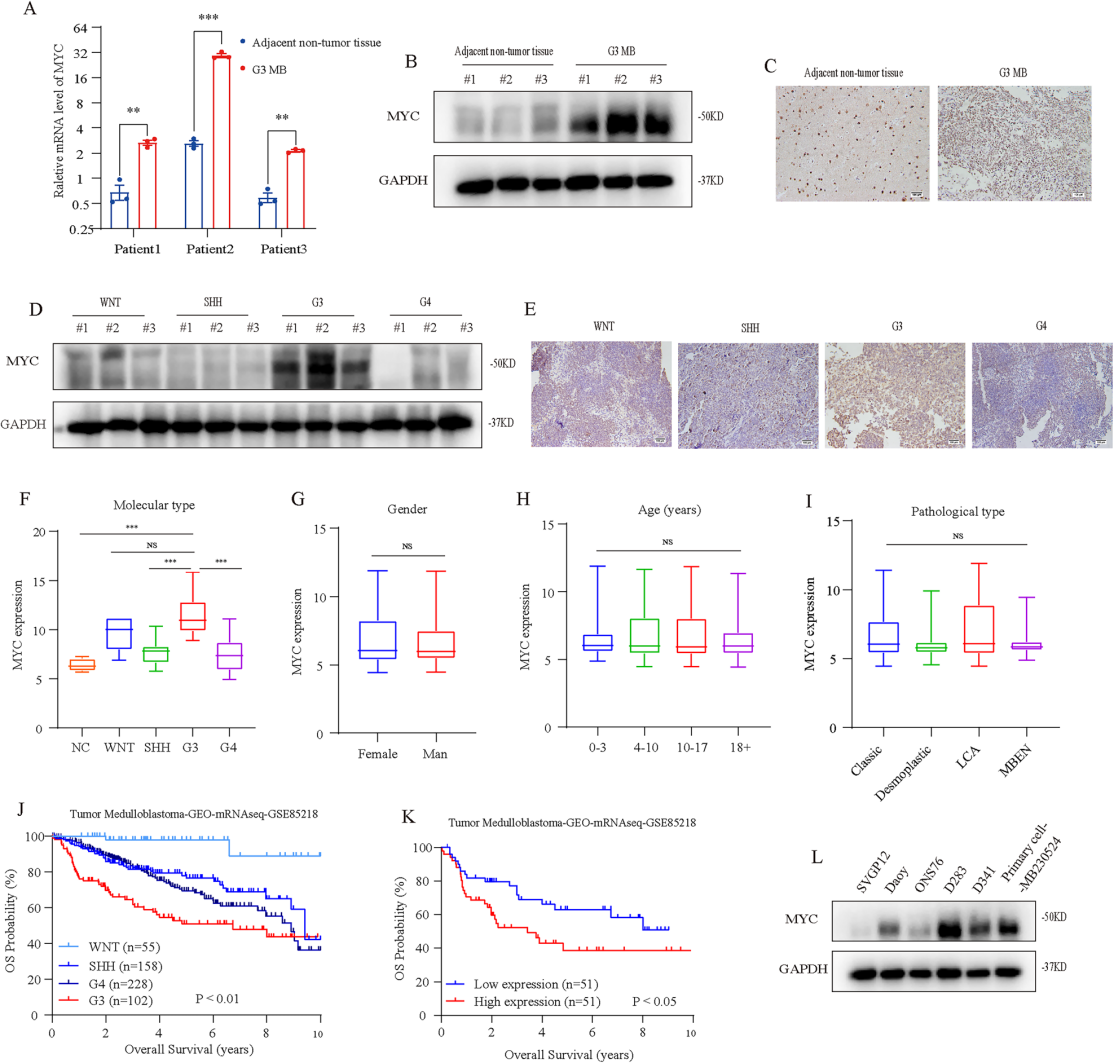
MYC is highly expressed in G3 MB and significantly correlated with prognosis. **A** qRT-PCR revealed an increase of MYC in the mRNA level in G3 MB. **B** Western blot was used to detect the expression levels of MYC in adjacent non-tumor tissues and G3 MB. **C** IHC staining of patient clinical MB sample and adjacent non-tumor tissue. **D** The expression levels of MYC protein in different subtypes. **E** IHC staining of MYC in WNT, SHH, G3 and G4 MB. **F** The expression level of MYC is highly correlated with molecular typing, with high expression in G3 type (GSE164677). **G-I** Correlation analysis between MYC expression level and different clinical features, including gender, age and conventional pathological type. **J** Results of the Kaplan-Meier analysis of progression-free survival in different subtypes MB patients (GSE85218). **K** Kaplan-Meier analysis of the association between MYC expression levels and overall survival of G3 MB patients. **L** Western blot was used to detect the relative expression levels of MYC in six cell lines (SVG p12, Daoy, ONS76, D283, D341 and MB230524). NS *p* > 0.05, *p < 0.05, **p < 0.01, and ***p < 0.001.

We obtained paraffin sections from the Pathology Department and frozen tumor tissue samples of MB patients from the hospital’s biological sample bank. Compared with adjacent non-tumor tissues, MYC is overexpressed in G3 MB tumor tissues (Fig. 1A–C). Because clinical prognosis is also highly correlated with methylation molecular type, we further examined the expression levels of MYC in different subtypes. MYC amplification was found in the G3 type but not in the SHH and G4 types (Fig. 1D, E), which is consistent with the expression data of MYC in the GEO database (GSE164677) (Fig. 1F). However, no significant correlation was found between MYC expression levels and different clinical features, including gender, age and conventional pathological type (Fig. 1G–I).

By further analyzing the prognosis of MB patients (total 543 cases) according to the GEO database (GSE85218), we confirmed the poor prognosis of G3 MB and determined the correlation of the MYC gene with overall G3 MB patient outcome (Fig. 1J, K). However, MYC expression levels showed no significant prognostic evaluation value in WNT and SHH subtypes (Supplementary Fig. 1A–C). Pearson correlation analysis of the GSE85217 dataset revealed significant associations between MYC and proteins involved in the HDAC/PI3K signaling pathway (*p* < 0.05). Specifically, MYC showed positive correlations with oncogenic factors HDAC2, HDAC3, AKT2, and AKT3, while demonstrating negative correlations with tumor suppressors PTEN and FOXO1 (Supplementary Fig. 1D-I). These findings suggest the potential feasibility of targeting the HDAC/PI3K pathway to regulate MYC expression in subsequent studies.

Finally, we screened out G3 MB cell (MB230524) from the patient-derived primary cells. DNA methylation result of this patient indicated G3 MB with MYC amplification, and MRI of whole brain and spinal cord showed tumor dissemination and metastasis (Supplementary Fig. 1J–L). Western blot was used to detect the expression of MYC, finding that the expression of MYC in D283 and MB230524 cells was higher than that in other cell lines (Fig. 1L). Therefore, we conducted subsequent experiments using D283 and MB230524 as representatives of MYC-amplified G3 MB.

### CUDC-907 can relatively safely inhibit the growth of MB cells by downregulating MYC

**Fig. 2:**
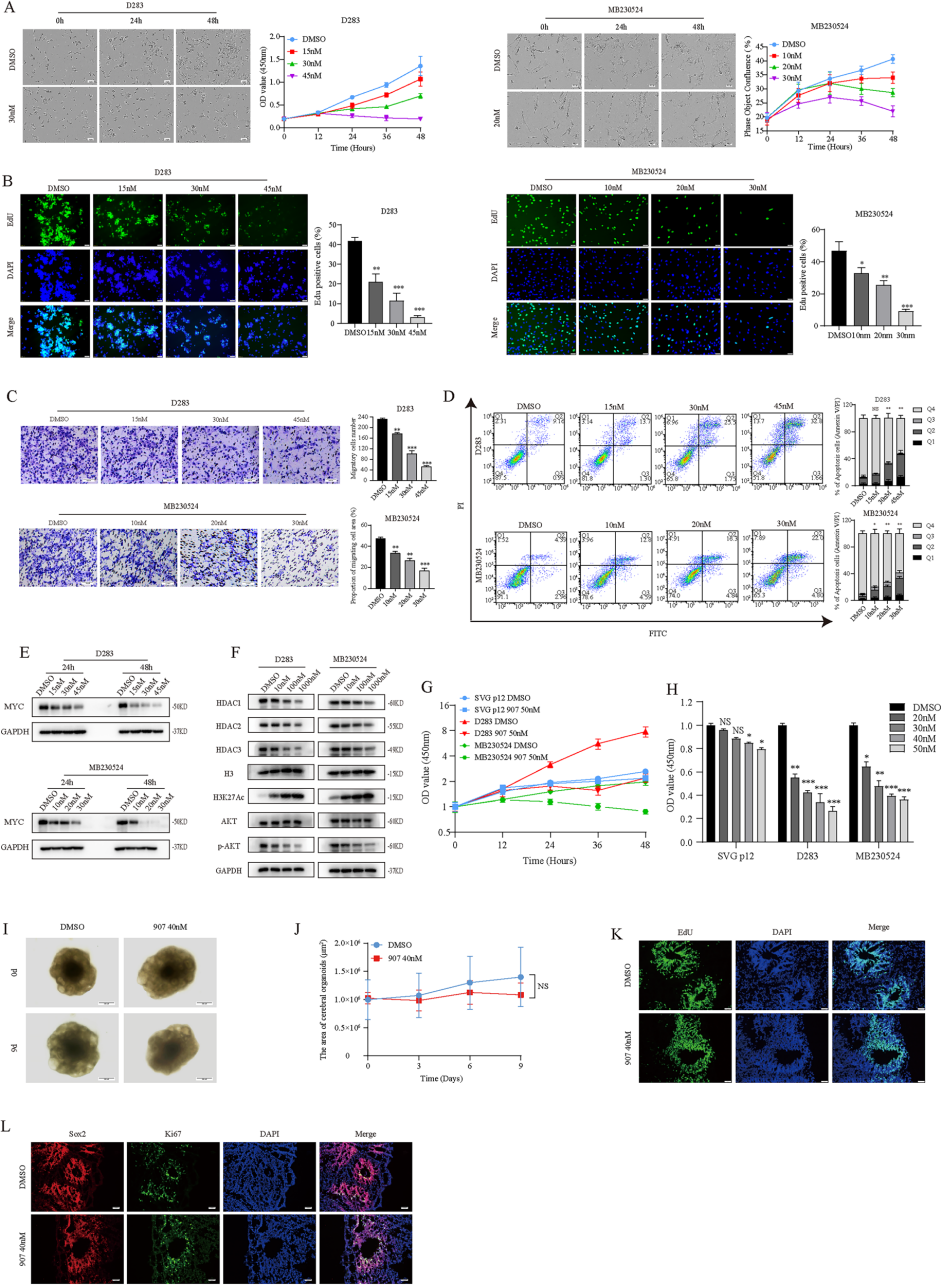
CUDC-907 can relatively safely inhibit the growth of MB cells by downregulating MYC. **A** Morphological images and growth curves of D283 and MB230524 were recorded after treatment of CUDC-907 for 48 h. **B** EdU assay was used to detect the effect of CUDC-907 on the DNA synthesis ability of MB cells. **C** The results of the transwell assay demonstrating the impact of CUDC-907 on the migratory capacity of D283 and MB230524 cells. **D** Apoptosis of MB cells treated with CUDC-907 was assessed by flow cytometry. **E** The expression of MYC was detected using western blot after treatment with CUDC-907 for 24 h and 48 h. **F** Western blot was used to detect the expression of HDAC and PI3K related proteins in the MYC upstream pathway. **G** Effect of CUDC-907 on proliferation rate of normal astrocyte SVG p12. **H** CCK8 assay was used to detect the viability of SVG p12 at the treatment endpoint. **I** Bright field images of cerebral organoids exposed to DMSO or 40 nM CUDC-907 for 9 days. **J** The 2D areas of three samples in each group were measured at 0 days, 3 days, 6 days and 9 days, and growth curves were plotted. **K** Results of the EdU assay in cerebral organoids. **L** Immunofluorescence (IF) analyses of cerebral organoids. NS *p* > 0.05, *p < 0.05, **p < 0.01, and ***p < 0.001.

When compared with commercially available HDAC inhibitor (Entinostat) and PI3K inhibitor (LY294002), CUDC-907 was more effective MB cell growth inhibitor with an half maximal inhibitory concentration (IC_50_) of 31.7 ± 2.8 nM and 17.6 ± 8.3 nM in vitro, which was at least 153-fold more potent than single pathway inhibitor (Supplementary Fig. 2A, B). After D283 and MB230524 were treated with CUDC-907 for 48 h, we observed that CUDC-907 inhibited the growth of MB cells in a concentration-dependent manner (Fig. 2A). The results of the EdU assay further revealed that CUDC-907 significantly inhibited the proliferation of MB cells by reducing DNA synthesis (Fig. 2B). Because G3 MB has strong invasive ability, transwell assay revealed that CUDC-907 significantly reduced the migration ability of MB cells (Fig. 1C). After treatment with CUDC-907, some dead cells were suspended in the culture medium. Therefore, Annexin V-FITC/PI kit was used to detect cell apoptosis. The flow cytometry revealed that CUDC-907 mainly caused premature apoptosis in MB cells (Fig. 1D). To explore whether the anti-MB activity of CUDC-907 is related to known pharmacological effects, we further evaluated the expression of MYC protein and the activity of the HDAC/PI3K upstream pathway, which are targets of CUDC-907. Western blot analysis revealed that CUDC-907 treatment downregulated MYC protein expression in MB cells (Fig. 2E). Concurrently, HDACs expression was reduced, accompanied by an increase in H3K27ac levels. Furthermore, the PI3K key factor phosphorylative-AKT decreased, indicating that the activity of PI3K was inhibited by CUDC-907 (Fig. 2F). These results revealed that CUDC-907 targets MYC through the HDAC and PI3K pathways, thereby inhibiting MB cells proliferation and invasion, and also inducing cell apoptosis.

In addition, because MB is one of the most common brain tumors in children, we further evaluated the safety of pediatric medication in normal astrocyte cell line SVG p12 and cerebral organoids. Compared with the MB cells, CCK8 results revealed that CUDC-907 had a limited effect on the growth of SVG p12 (Fig. 2G, H). Moreover, through the induction and differentiation of human embryonic stem cells (hESCs), we have established cerebral organoids, which are utilized to simulate “normal brain tissue” to test drug toxicity. The results revealed that CUDC-907 had no remarkable effect on the morphology and proliferation of cerebral organoids (Fig. 2I, J). No significant changes were found in the corresponding molecular characteristics, including the DNA synthesis marker EdU, the proliferation marker Ki67 and the stem cell marker Sox2 (Fig. 2K, L). Taken together, these results suggested that normal brain tissue has good tolerance to CUDC-907 and subsequent in vivo experiments are feasible.

### CUDC-907 disrupts MYC to regulate the transcription of P21 and P27, then resulting in G0/G1 phase arrest of the cell cycle

**Fig. 3:**
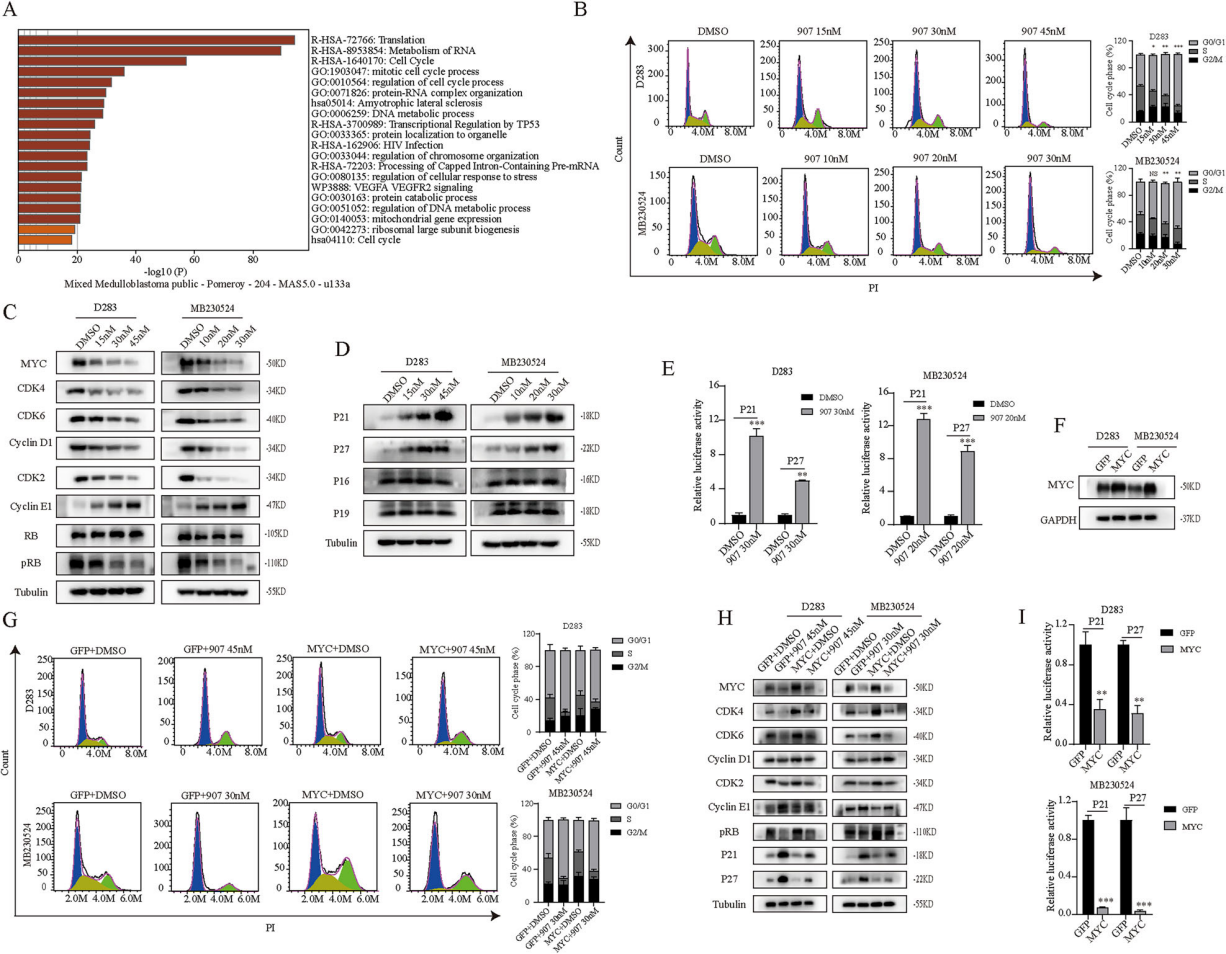
MYC exerts G0/G1 phase arrest via the transcriptional regulation of P21 and P27. **A** Data mining according to R2 Genomics Analysis and Visualization Platform revealed that MYC is closely related to the cell cycle. **B** Flow cytometry analysis revealed that the proportion of G0/G1 phase among MB cells treated with CUDC-907 increased with increasing drug concentration. **C** Western blot was used to detect the expression levels of G0/G1 related cyclin-dependent kinases (CDKs) and cyclins. **D** The expression of upstream CDK inhibitors (CKIs). **E** CUDC-907 enhanced the fluorescence activity of plasmids carrying the P21 and P27 promoters. **F** MYC was overexpressed in MB cells. **G** Overexpression of MYC weakened the G0/G1 phase blockade induced by CUDC-907. **H** Western blot detection of G0/G1 phase protein level. **I** Dual-luciferase assays revealed a relative decrease in the fluorescence activity of P21 and P27 after the overexpression of MYC. NS *p* > 0.05, *p < 0.05, **p < 0.01, and ***p < 0.001.

Previous studies have shown that downregulation of MYC protein is an early event induced by CUDC-907 therapy. However, MYC protein is multifunctional, and it remains unclear which specific functional alterations are induced by its downregulation. Through R2 Genomics Analysis and Visualization Platform data mining (Mixed Medulloblastoma Public Pomeroy 204 MAS 5.0-u133a), MYC was found to be closely related to the cell cycle in addition to conventional transcriptional regulation and RNA metabolism (Fig. 3A). Following flow cytometry analysis, CUDC-907 significantly induces G0/G1 cell cycle arrest in MB cells (Fig. 3B). It is known that cyclins and cyclin-dependent kinases (CDKs) jointly regulate cell cycle progression. The cyclin D-CDK4/6 and cyclin E-CDK2 complexes are crucial for the G1/S transition. When they are inhibited, the cell cycle will stop at the G0/G1 phase. Both of these complexes can phosphorylate RB, causing the RB-E2F complex to release the transcription factor E2F and increase the expression of downstream target genes [25]. Therefore, we detected the relevant proteins mentioned above and found that CUDC-907 inhibited the expression of CDK2, CDK4, CDK6, cyclin D1, and pRB in a concentration-dependent manner while increasing the expression of cyclin E1 (Fig. 3C).

There is a brake system for CDK inhibitors (CKIs) in the cell cycle to prevent unrestricted cell proliferation. These small peptide inhibitors can directly bind to CDKs and inactivate them [25]. Ink4 and Cip/Kip are the two main families of CKIs. Members of the Ink4 family, including p16Ink4a, p15Ink4b, p18Ink4c and p19Ink4d, bind only to CDK4 and CDK6. Members of the Cip/Kip family, including p21Waf1/Cip1, p27Kip1 and p57Kip2, exhibit broader specificity in binding to cyclin D-CDK4/CDK6, cyclin E-CDK2, cyclin A-CDK2 and cyclin B-CDK1. Further western blot analysis revealed that CUDC-907 had no significant effect on the expression of P16 or P19, but significantly increased the expression of P21 and P27 (Fig. 3D). This finding also explained why CUDC-907 can cause changes in other cyclins and CDKs in addition to CDK4 and CDK6 (Fig. 3C). Furthermore, dual-luciferase reporter assays confirmed that, compared with the control group, treatment with the IC_50_ concentration CUDC-907 for 48 h significantly increased the fluorescence activity of the PGL3 plasmids carrying the P21 and P27 promoters (Fig. 3E). These results suggest that CUDC-907-mediated downregulation of MYC expression attenuates the transcriptional regulation of CKIs, leading to an increase in the expression of P21 and P27, thereby inhibiting the corresponding cyclins and CDKs, which eventually leads to G0/G1 phase arrest and exerts anti-tumor effects.

To investigate whether this inhibition was occasioned by an off-target effect, we overexpressed MYC in MB cells (Fig. 3F). Flow cytometry analysis revealed that overexpression of MYC weakened the G0/G1 arrest induced by CUDC-907 (Fig. 3G). MYC amplification also rescued the downregulation of CDK2, CDK4, CDK6, cyclin D1, and pRB, while reversing the upregulation of cyclin E1, P21, and P27 (Fig. 3H). Finally, dual-luciferase reporter assays revealed that overexpression of MYC significantly reduced the relative fluorescence activity by inhibiting transcription of P21 and P27 (Fig. 3I). These findings indicated that CUDC-907 indeed induces G0/G1 phase arrest in MB cells through the transcriptional regulation function of MYC to exert antitumor effects.

### CUDC-907 sensitizes MB cells to chemotherapy and radiotherapy

**Fig. 4:**
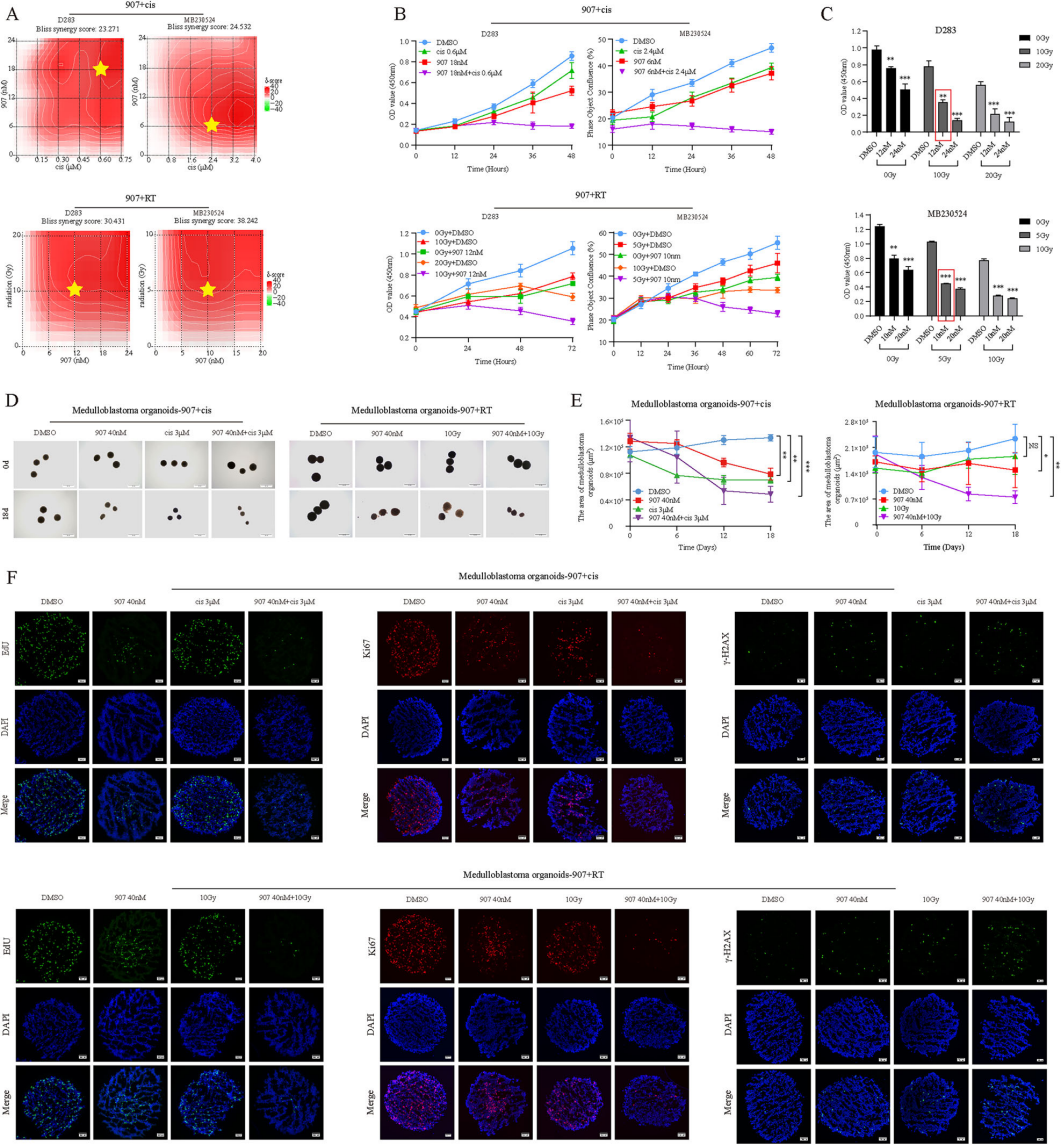
CUDC-907 enhances MB sensitivity to chemoradiotherapy. **A** SynergyFinder online tool was used for bliss synergy analysis to evaluate the synergistic effect of the combination treatment, with an average bliss synergy score greater than 10. **B** IncuCyte cell confluency and CCK8 assays revealed that CUDC-907 + cisplatin or CUDC-907 + RT had synergistic inhibitory effects on cell proliferation. **C** Low-dose CUDC-907 combined with RT significantly inhibited cell viability, and its effect was even greater than that achieved by doubling the radiation dose alone. **D** Bright field image diagrams of MBOs before and after combination therapy. **E** Quantification of the relative MBOs size over time. **F** The EdU and IF staining results of MBOs after one week of treatment. NS *p* > 0.05, *p < 0.05, **p < 0.01, and ***p < 0.001.

At present, chemotherapy and radiotherapy (RT) remain the primary adjuvant therapies following MB surgery. High-dose chemotherapy and RT are often needed for very high-risk MB, which undoubtedly increase the incidence of toxic side effects and reduce quality of life. Therefore, finding effective sensitizers for chemotherapy and RT has great clinical significance. Because cisplatin is the most common chemotherapeutic drug for MB [26–28], the combination of CUDC-907 and cisplatin or RT was tested in this study. At the endpoint of treatment, the OD value corresponding to cell viability was analyzed via the SynergyFinder online tool. The combination treatment synergistically reduced the number of viable MB cells across a range of doses with average bliss synergy scores greater than 10 (Fig. 4A). After the appropriate combination scheme was selected according to the bliss synergy scores, IncuCyte cell confluency and CCK8 assays were used to record the synergistic effects of CUDC-907 + cisplatin and CUDC-907 + RT on MB cells proliferation (Fig. 4B). Notably, the combination of 12 nM CUDC-907 and 10 Gy irradiation significantly inhibited the viability of D283 cells, and the level of inhibition was even greater than that achieved by 20 Gy irradiation alone (Fig. 4C). Similar results can also be observed in MB230524 cells. These data indicated that CUDC-907 is expected to reduce the dosage of chemotherapy and RT while ensuring treatment effectiveness.

To better align with clinical practice, we extracted MBOs from the fresh tumor tissue of G3 MB patients with MYC amplification (Supplementary Fig. 1K). The use of VX-970 and Entinostat was recommended, as ATR and HDAC inhibitors respectively, both of which have the ability to regulate downstream MYC [29–31]. Because CUDC-907 has similarities with them and MBOs resembles human parental tumors in terms of both histology and gene expression patterns, CUDC-907 is a theoretically effective therapy. The growth curve revealed that MBOs are highly sensitive to CUDC-907 (Supplementary Fig. 3A). For better comparison with the cerebral organoids data shown in Fig. 2, the administration concentration of CUDC-907 was also set to 40 nM. Furthermore, when CUDC-907 is used in combination with cisplatin or RT, the volume of MBOs significantly decreased with irregular shapes, rough edges, brittle textures and increased tissue fragments (Fig. 4D). According to the 2D area plot, the inhibitory effect of CUDC-907 combined with cisplatin or RT gradually increased over time, surpassing that of any single drug (Fig. 4E). In addition, compared with those in the control group, there was a significant decrease in EdU and Ki67, and an increase in the DNA damage marker γ-H2AX after drug treatment (Fig. 4F). In summary, we demonstrated from both micro- and macro-perspectives that CUDC-907 combined with chemotherapy or RT has the potential to improve the treatment outcomes of MYC-driven G3 MB patients.

### CUDC-907 + cisplatin and CUDC-907 + RT inhibit MB activity through different mechanisms

**Fig. 5:**
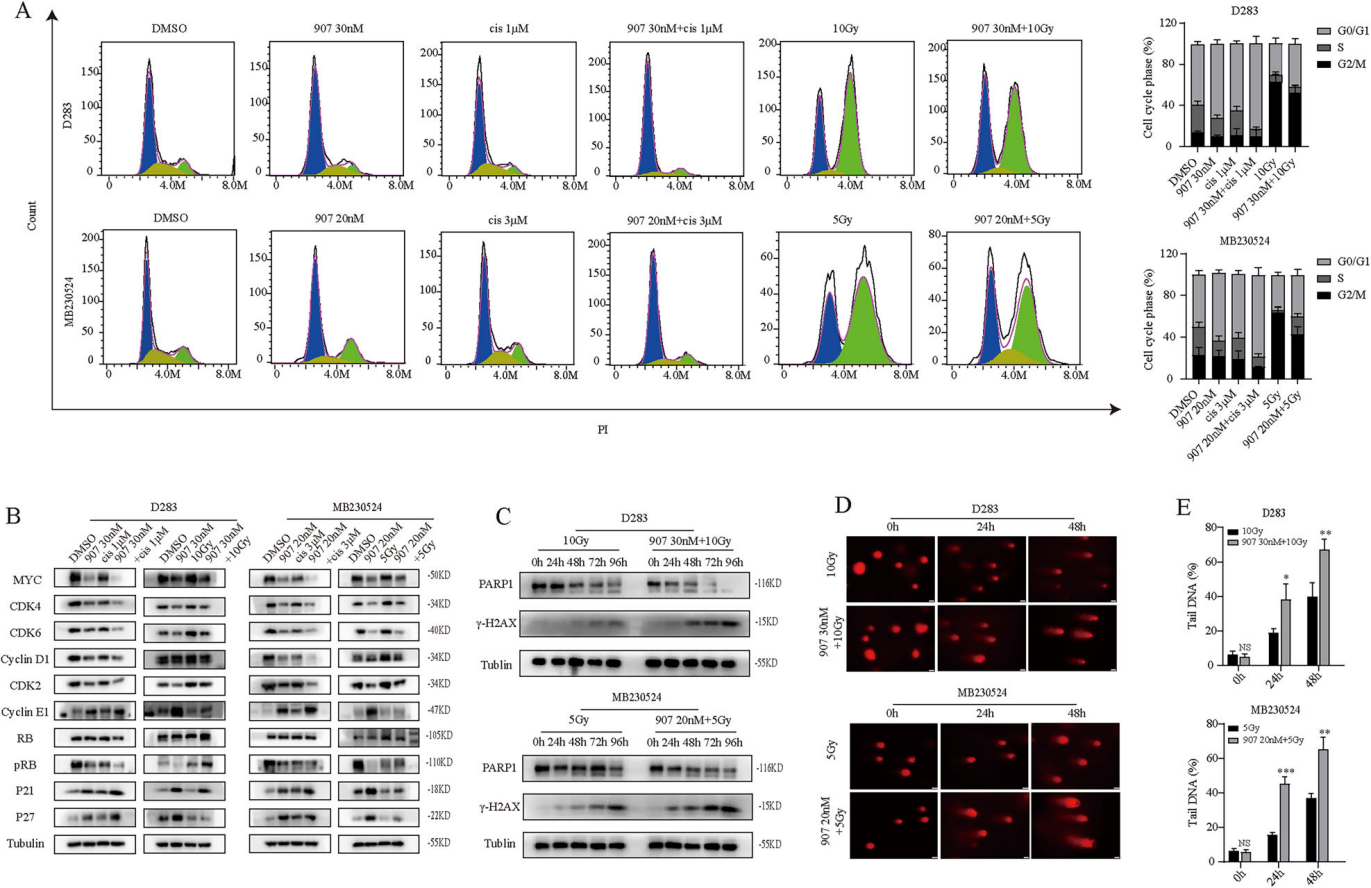
Mechanisms related to combination therapy. **A** Flow cytometry analysis of D283 and MB230524 treated with combination therapy. **B** Expression levels of G0/G1 phase related proteins after combination therapy. **C** Expression levels of DNA repair marker PARP1 and DNA damage marker γ-H2AX. **D** A single-cell alkaline comet assay was used to observe the degree of DNA damage. **E** The tail moments of 10 cells in each group were tested, and a bar chart was drawn. NS *p* > 0.05, *p < 0.05, **p < 0.01, and ***p < 0.001.

Figure 3 showed that CUDC-907 exerts antitumor effects by inducing G0/G1 phase arrest, but it is currently unknown whether combination therapy enhances this function. After pretreatment with CUDC-907 for 12 h, MB cells were treated with cisplatin or RT. Flow cytometry cycle analysis at 48 h revealed that both CUDC-907 and cisplatin could increase G0/G1 phase arrest when used alone. Importantly, this effect was further enhanced when they were combined. However, the combination of CUDC-907 and RT did not seem to have a synergistic effect on the cell cycle and even showed opposite trends (Fig. 5A). RT was different from CUDC-907, mainly leading to G2/M blockage, which was consistent with previous articles [32, 33]. Interestingly, the G2/M phase arrest was partially reversed when RT was used with CUDC-907. The G2/M phase ratio of D283 cells decreased from 68.7 ± 7.8% to 52.5 ± 6.5%, and that of MB230524 also decreased from 63.5 ± 5.8% to 42.4 ± 7.6%. Western blot analysis also revealed that the expression of MYC, CDK2, CDK4, CDK6, cyclin D1 and pRB in CUDC-907 + cisplatin group was lower than that in only CUDC-907 or cisplatin group, and the expression of cyclin E1, P21 and P27 further increased. Similarly, this trend was not observed in the MB cells treated with the combination of CUDC-907 and RT (Fig. 5B). The above results suggested that CUDC-907 + cisplatin exert better anti-MB effects by enhancing G0/G1 phase arrest, whereas CUDC-907 + RT may exert synergistic effects through other potential mechanisms.

Previous studies have shown that DNA double-strand breaks are the most lethal damage induced by irradiation and can trigger a series of cellular DNA damage responses (DDRs), including the activation of DNA damage sensing and early transduction pathways, cell cycle arrest, and DNA repair. Because G2/M arrest allows DNA damage repair after RT, we anticipated that the combination of CUDC-907 and RT would inhibit DNA damage repair and increase the degree of DNA damage [34–36]. As expected, after pretreatment with CUDC-907 for 12 h, D283 and MB230524 were irradiated with 5 Gy and 10 Gy respectively, and the cell proteins were extracted at 0 h, 24 h, 48 h, 72 h, and 96 h. The western blot results revealed that the combination of CUDC-907 and RT resulted in an earlier and greater decrease in the DNA repair marker PARP1 than RT alone. Moreover, the expression level of the DNA fragmentation marker γ-H2AX correspondingly increased (Fig. 5C). Single-cell alkaline comet assays confirmed that the combination therapy group presented more obvious comet tails at both 24 h and 48 h (Fig. 5D, E). These findings indicated that the enhanced efficacy of CUDC-907 in combination with RT is due not only to abrogation of G2/M arrest but also to direct effects on the DNA damage repair machinery, resulting in a relative increase in the degree of DNA damage.

### CUDC-907 inhibits MB cells tumorigenesis and sensitizes MB cells to cisplatin and RT in vivo

**Fig. 6:**
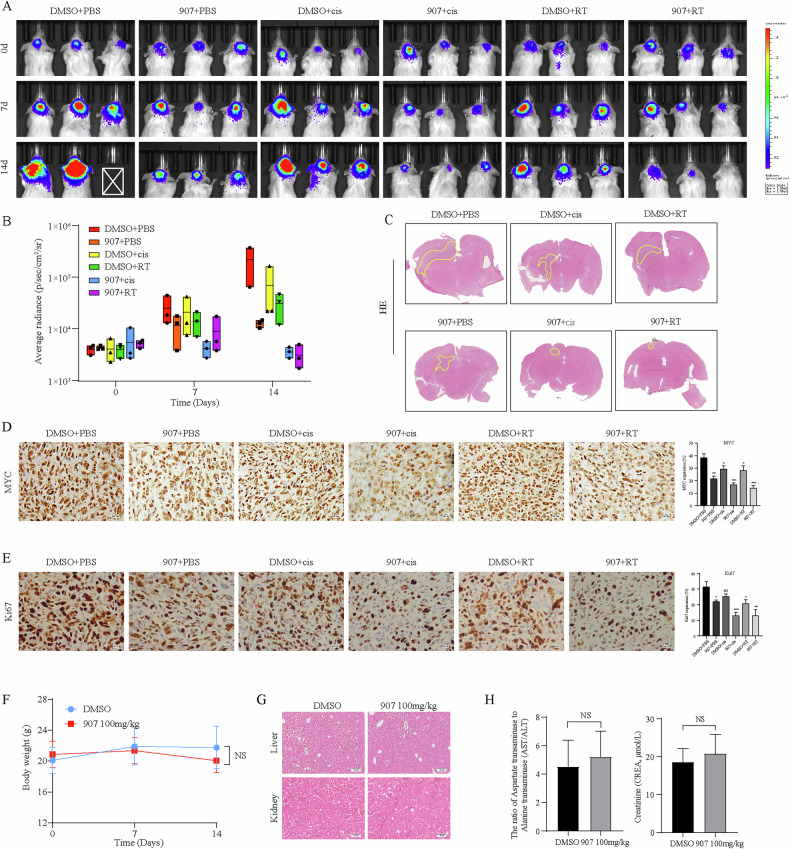
CUDC-907 inhibits the growth of MB in intracranial xenograft models. **A** IVIS images of the mice before and after treatment. **B** A growth curve was drawn on the basis of the measured average radiance value. **C** HE staining of intracranial tumors at the treatment endpoint, with yellow lines indicating the tumor areas. **D** IHC analyses of MYC protein expression in xenografts mice. **E** IHC analyses of Ki67. **F** Body weight curves corresponding to the DMSO group and the 100 mg/kg CUDC-907 group. **G** HE staining results of mouse liver and kidney. **H** Results of liver and kidney function testing, including AST/ALT and CREA. NS p > 0.05.

We further investigated the potential therapeutic efficacy of combination treatment using intracranial orthotopic xenograft models. For the human MB D283 xenograft model, visible tumors were detected by IVIS imaging as early as 7 days post-injection. Following tumor verification, the mice were treated with DMSO, CUDC-907, cisplatin alone, RT alone, CUDC-907 + cisplatin or CUDC-907 + RT to simulate a patient analogous fractionation schedule. Tumors size, as measured by IVIS signal, was visibly reduced within two weeks after combination treatment, that was consistent with previous results of MB cells and MBOs (Fig. 6A, B). To further validate our findings in vivo, HE staining revealed that the tumor volume marked by the yellow line was markedly decreased (Fig. 6C). Moreover, IHC analysis of MYC and Ki-67 revealed that combination treatment suppressed cell proliferation by suppressing MYC (Fig. 6D, E).

In terms of safety, the mice that were administered 100 mg/kg CUDC-907 once a day did not exhibit significant weight loss (Fig. 6F). According to HE staining, the liver and kidney of the CUDC-907 group had clear tissue structures, and no pathological changes were found (Fig. 6G). We also collected blood samples to assess liver and kidney functions, and no statistically significant differences were observed in the ratios of aspartate transaminase to alanine transaminase (AST/ALT) and creatinine (CREA) (Fig. 6H). Together, these data established CUDC-907 as a safe cisplatin or RT sensitizer in MB xenograft models.

## Discussion

Given that the MB molecular subgroup characterized by MYC copy number amplification is associated with significantly worse event-free and overall survival outcomes, and conventional therapies often fail to achieve a cure for these patients, numerous preclinical studies have explored potential therapeutic strategies for high-risk MB. These include combination therapy with PI3K pathway inhibitors (BKM-120) and histone deacetylase inhibitors (LBH-589) for MYC-driven group 3 medulloblastoma, targeting LSD1 in MB with GFI1/GFI1B over-activation, CDK inhibitors, and cell cycle checkpoint inhibitors [37–39]. In this study, CUDC-907 possesses potent antitumor abilities by integrating multiple similar mechanisms. Unlike previous studies, which used MB cell lines that had undergone several passages in culture, we comprehensively elucidated the antitumor activity of CUDC-907 in primary cells, MBOs freshly isolated from human tumor tissue and animal models. Furthermore, we have explored the important role of the HDAC/PI3K-MYC-P21/P27-CDKs/cyclins-G0/G1 phase axis in the anti-MB activity. Finally, the combination of CUDC-907 and cisplatin can further enhance G0/G1 phase arrest to achieve better therapeutic effects. In terms of RT, CUDC-907 can facilitate the abrogation of RT-induced G2/M phase arrest, which resulted in a diminished capacity of MB cells treated with CUDC-907 to repair radiation-induced DNA damage. Our current research has shown that CUDC-907, as a potential cisplatin or RT sensitizer, may be a new treatment concept for G3 MB patients.

At present, a phase I clinical trial (NCT02307240) is underway to evaluate the safety and pharmacokinetics of CUDC-907 in patients with advanced or recurrent solid tumors. For pediatric tumors, an active clinical trial (NCT03893487) is investigating the use of CUDC-907 in treating brain tumors in children and young adults. CUDC-907 has demonstrated a moderate safety profile, with reported side effects primarily limited to mild gastrointestinal and hematologic adverse events (NCT02909777) [40, 41]. Our study also evaluated the safety of CUDC-907 from the aspects of normal astrocyte SVG p12, cerebral organoids and animal tolerance, providing a basis for the entry of CUDC-907 into MB clinical trials.

Unlike poorly specific 2D cell models or time-consuming animal models, previous studies have shown that tumor organoid models may be more suitable for rapidly studying patient-specific treatment strategies [42]. In addition, traditional tumor cultures clone and select highly proliferating cells in growth factor-rich media, reducing the proportion of slowly proliferating and non-proliferating cells originally present in parental tumors, which may play an important role in the recurrence and drug resistance of tumors [43]. In this study, we conducted vitro experiments by using MBOs derived from metastatic G3 MB patients with MYC amplification, that avoids single-cell dissociation and preserves native cell-cell interactions. We found that CUDC-907, especially when combined with chemotherapy and RT, significantly inhibited the proliferation of MBOs. Of course, more clinical specimens and trials are needed to verify these findings, and our current MBOs drug screening platform will provide support for this.

CUDC-907, as a dual-function monotherapy, also has limitations, which may lose the flexibility in independently adjusting the dosage. However, this type of bifunctional candidate drug has simpler clinical development process than combination therapy, because its pharmacokinetics and toxicity profile are easier to predict and manage. Furthermore, considering the pleiotropy of HDAC inhibition and the multiple downstream effects of PI3K, the role of CUDC-907 may not be attributed only to MYC inhibition. Further research is needed to analyze the primary and secondary effects of CUDC-907 on the oncogenic network and identify other sensitive tumor types.

In summary, this study revealed that CUDC-907 effectively inhibits MYC, which may benefit G3 MB patients with MYC overexpression. CUDC-907 also sensitizes MB cells, organoid and xenograft models to chemotherapy and radiotherapy. On the basis of promising preclinical research results, this new treatment approach in turn could impact future clinical investigations for this patient population.

## Materials and methods

### Cell lines, antibodies, and reagents

This study established MB primary cell lines and MB organoids (MBOs) from freshly excised tumor. We screened for MYC-amplified G3 type case (MB230524) based on the patient’s DNA methylation result. The Magnetic Resonance Imaging (MRI) of whole brain and spinal cord showed tumor dissemination and metastasis (Supplementary Fig. 1J–K). Human-derived MB Daoy cells (RRID: CVCL_1167), ONS-76 cells (RRID: CVCL_1624), D283 cells (RRID: CVCL_1155) and D341 cells (RRID: CVCL_0018) were purchased from ATCC Corporation in China. Daoy cells were cultured in Dulbecco’s modified Eagle’s medium (DMEM, Gibco, USA). ONS-76 cells were cultured in 1640 medium (Gibco, USA). D283, D341 and MB primary cells (MB230524) were cultured in Eagle’s minimum essential medium (MEM, Gibco, USA). They were supplemented with 10% (v/v) fetal bovine serum (FBS, HyClone, USA) and stored in a 37 °C humidified environment containing 5% CO2. The c-myc (#10828-1-AP), HDAC1 (#10197-1-AP), HDAC2 (#12922-3-AP), HDAC3 (#81211-1-RR), H3 (#17168-1-AP), H3K27AC (#82902-1-RR), GAPDH (#10494-1-AP), CDK2 (#10122-1-AP), cyclinE1 (#11554-1-AP), P16 (#10883-1-AP), P19 (#10272-2-AP), P21 (#10355-1-AP), P27 (#25614-1-AP), PARP1 (#13371-1-AP) and Tubulin (#14555-1-AP) antibodies were purchased from Proteintech (Wuhan, China). The anti-SOX2 (#ab79351) antibody was purchased from Abcam (Shanghai, China). The AKT (#9272 s), phospho-Akt (#4060 s) and γ-H2AX (#9718 T) antibodies were purchased from Cell Signaling Technology (Beverly, MA, USA). CDK4 (#sc-56277) and CDK6 (#sc-53638) antibodies were purchased from Santa Cruz (Dallas, TX, USA). The cyclin D1 (#A19038), RB (#A16966) and p-RB (#AP0484) antibodies were purchased from ABclone (Wuhan, China). The cycle detection kit (#C1052), EdU detection kit (#C0078S), comet assay kit (#C2041S) and gentian violet (#C0121) were purchased from Beyotime Biotechnology (Shanghai, China). Dimethyl sulfoxide (DMSO, #D2650) were purchased from Sigma-Aldrich (USA). The apoptosis detection kit (#40302ES60) and dual luciferase assay kit (#11402ES60) were purchased from Yeasen (Shanghai, China). CUDC-907 (#S2759), PEG300 (#S6704) and Tween 80 (#S6702) were purchased from Selleck (Houston, TX, USA). CUDC-907 was dissolved in DMSO to generate a 10 mM stock solution. The samples were stored at −80 °C for in vitro studies. For in vivo studies, CUDC-907 was dissolved in 5% DMSO–40% PEG300–5% Tween 80–50% ddH2O.

### Transfection and infection

The GFP-specific overexpression fragment and MYC-specific overexpression fragment (NM_002467) were subsequently cloned and inserted into the pcDNA3.1-T2A-EGFP2 vector. The P21 (NM_000389) and P27 (NM_004064) promoter sequences were ligated into the pGL3-Basic vector. The above vectors were obtained from Youbao Corporation (Changsha, China). The procedures used for transfection and infection were previously described [44].

### Western blot analysis

The cells and tissues were lysed with RIPA and PMSF (Beyotime Biotechnology). The proteins were separated on 8–12% SDS–PAGE gels and transferred to PVDF membranes. After being blocked with 5% BSA solution, the membranes were incubated with primary antibodies overnight at 4 °C and then with HRP-linked secondary antibodies for 1 h at room temperature. The membranes were exposed by a VIBER FUSION FX6 EDGE (Paris, France).

### Quantitative RT–PCR

Total RNA was harvested from MB tumor tissue with Trizol reagent, and then the concentration of total RNA was subsequently measured. Two micrograms of RNA was reverse transcribed into cDNA with a PrimeScript™ RT reagent Kit with gDNA Eraser (#RR047A, Takara). The expression of mRNA was measured by using a Bio-Rad CFX Opus 96 Real–Time PCR System. The △△Ct method was applied for qRT–PCR analysis. The primers used for qRT–PCR assays included c-myc-F (TGGAAAACCAGCCTCCCG), c-myc-R (TTCTCCTCCTCGTCGCAGTA), GAPDH-F (CAGGAGGCATTGCTGATGAT), and GAPDH-R (GAAGGCTGGGGCTCATTT).

### Cell proliferation assay

The proliferation rate of MB cells was detected via CCK8 assays and IncuCyte® cell confluence assays. To test the viability of the cells, we cultured 2000 cells/well in a 96-well plate for 48 h or 72 h. As semiadherent and semisuspended cells, the viability of D283 cells was measured via a CCK8 kit (#SB-CCK8-100 ml, share-bio) every 12 h. The IncuCyte ZOOM (Sartorius, Germany) monitored MB230524 cells growth every 12 h by using the integrated confluence algorithm as a surrogate for cell number.

### 5-Ethynyl-2′-deoxyuridine (EdU) assay

The DNA synthesis ability of MB cells was evaluated via a BeyoClick™ EdU Cell Proliferation Kit with Alexa Fluor 488 (#C0071S, Beyotime). The procedure was performed according to the manufacturer’s instructions. Notably, we digested and collected all D283 cells before conducting EDU assays due to the suspended growth of some D283 cells. After EdU staining, cells were visualized via the fluorescence microscope, and the percentage of EdU-positive cells was calculated as the number of EdU-positive cells out of the total number of cells (× 100).

### Migration assay

Transwell chambers were used to detect cell migration. MEM supplemented with 10% FBS as an inducer was placed under the chamber, and 4 × 10^4^ cells cultured with 1% FBS MEM were placed in the chamber. After being cultured for 8 h, the cells were fixed with 4% PFA for 15 min and stained with gentian violet for 10 min. At least 5 areas were selected for imaging under a microscope.

### Flow cytometry

For cell cycle detection, the indicated cells were harvested and fixed in 75% ethanol at 4 °C for 24 h. The cell lysate was washed with PBS buffer, stained with RNase A and propidium iodide (#C1052, Beyotime) at 4 °C for 30 min, and then analyzed via the CytoFLEX flow cytometer (BECKMAN, USA). For detection of cell apoptosis, the indicated cells were harvested and resuspended in binding buffer, followed by Annexin-V-FITC and PI staining with an Annexin-V-FITC/PI Apoptosis Detection Kit (#40302ES60, Yeasen).

### Dual-luciferase reporter assay

The PGL3-Basic with GFP and pRL-TK vectors were directly cotransfected into D283 and MB230524 as negative controls. The PGL3-Basic vectors with the P21/P27 promoter and pRL-TK vectors were cotransfected into D283 and MB230524, which have been transiently overexpressing MYC or pretreated with CUDC-907 for 12 h, as the experimental group. After 36 h, the dual luciferase reporter assay was performed according to the manufacturer’s instructions (#11402ES60, Yeasen).

### Comet assay

The comet assay was performed following the manufacturer’s protocol (#C2041S, Beyotime). The tail moment was quantitated in at least 10 independent cells via Adobe Illustrator software.

### MB organoids (MBOs) processing and immunofluorescence (IF)

MBOs were distributed in ultralow attachment 6-well culture plates (Corning) with 4 mL MBO medium and placed on an orbital shaker rotating at 120 rpm at 37 °C, 5% CO2, and 90% humidity sterile incubator. Approximately 75% of the medium was changed every 48 h. After drug treatment, some MBOs were incubated with 1 × EdU solution overnight for subsequent EdU assays according to the manufacturer’s instructions. Other MBOs were fixed overnight in 4% methanol-free formaldehyde and stored in 30% sucrose in formaldehyde at 4 °C until processing. Serial tissue sections were sliced via a cryostat (CM3050S, Leica) and melted onto charged slides (Thermo Fisher Scientific). The slides (6 μM) were dried at 60 °C for 30 min and stored at −40 °C until immunohistology.

For IF staining, the tissue sections were transferred to antigen repair solution (#C1038, Solarbio) at 95 °C for 30 min and then returned to room temperature naturally. The tissue sections were blocked with 5% BSA at room temperature for 1 h and incubated with primary antibodies including Ki67 (#MA5-14520, Invitrogen, 1:200) and γ-H2AX (#9718 T, CST, 1:200), overnight at 4 °C. On the second day, the tissue sections were incubated with secondary antibodies for 2 h at room temperature. After being stained with DAPI and quenched with anti-fluorescence, the slices were sealed for observation.

### Generation of cerebral organoids

We performed digestion and plating when the degree of human embryonic stem cells (hESCs) fusion in the six-well plate reached 80%-85%. The cells were diluted at a 1:10 ratio in a new six-well plate coated with Vitronectin (VTN-N, #A14700, CST). Essential 8 medium (#A1517001, CST) containing ROCK inhibitor Y27632 (#SCM075, Millipore) was added to each well, and the culture was continued at 37 °C in a 5% CO2 incubator. When the degree of cell fusion reached 70% -80%, hESCs were digested with TrypLE (#12604013, CST) and inoculated into a 96-well U-plate at a density of 9000 cells/well. The bFGF (#100-18B, Peprotech) and Y27632 was added to hESCs media for further cultivation. Six days later (D6), the generated embryoid bodies (EBs) were transferred to a low-adhesion 24-well plate, and the culture was continued with neural induction media (NIM). We coated EBs with Matrigel and changed the culture medium to cerebral organoid differentiation media (CODM) on D12. Furthermore, cerebral organoid maintenance media (COMM) was used, and the EBs were transferred to a horizontal shaker at a speed of 80 r/min on D16. At this time, we added CUDC-907 to the culture medium and recorded the morphology and size of the cerebral organoids. The cerebral organoids were collected for EdU and IF staining after adding CUDC-907 for 9 days. The components of each culture medium (including hESCs media, NIM, CODM and COMM) and the detailed cultivation methods were reference to a report [45].

### Xenograft assay

Six-week-old female NOD-SCID mice were purchased and housed in an SPF room that was maintained at a constant temperature and humidity. Human D283 cells (1 × 10^6^ cells) stably expressing luciferase were injected slowly into mouse skull. After 7 days of intracranial implantation, 150 mg/kg D-fluorescein (#HY-125918, MCE) was intraperitoneally injected, followed by bioluminescence imaging detection via the IVIS2000 system (Perkin Elmer IVIS Spectrum BL). The mice whose signals were detected were randomly divided into 6 groups (*n* = 3): the control group, the cisplatin group, the CUDC-907 group, the CUDC-907 + cisplatin group, the RT group, and the CUDC-907 + RT group. CUDC-907 was administered orally at 100 mg/kg for 10 days (from Monday to Friday, 2 weeks). Cisplatin was injected intraperitoneally at a dose of 2 mg/kg twice a week for a total of 2 weeks. Radiation was administered at 1.0 Gy every other day (Monday-Wednesday-Friday, 2 weeks), for a total dose of 6.0 Gy. Imaging was performed on the 14th and 21st days after administration. On the 21st day, the mice from each group were euthanized by inhaling isoflurane, and their brain tissues were collected. HE staining and IHC analyses were performed to confirm the histology and proliferation of the tumor cells. HE staining was also performed on mouse liver and kidney tissues, and blood was collected to test liver and kidney function. All studies were approved by the Animal Care and Use Committee of Jinfeng Laboratory (No.IACUC-JFLAB) and were carried out in accordance with the Guide for the Care and Use of Laboratory Animals (Ministry of Science and Technology of China, 2006).

### Immunohistochemistry (IHC) and hematoxylin–eosin (HE) staining

For each type of medulloblastoma (WNT, SHH, G3, and G4), we randomly selected 3 cases and obtained their paraffin sections from Pathology Department. IHC and HE staining were performed according to previous methods [46].

### Patient data analysis and patient tumor tissues

MB patient datasets were analyzed via publicly available patient databases from the R2 Genomic Analysis and Visualization Platform. Patient data and clinical MB samples were obtained from the Children’s Hospital of Chongqing Medical University. Tissue analysis was allowed by the Children’s Hospital of Chongqing Medical University (No.2024312). Written informed consent to participate was provided by the children (>8 years) or their families.

### Statistical analysis

All experiments were carried out in triplicates, and the quantitative data were expressed as the means ± SDs. One approach is to use the *t*-test to calculate the significance at the 95% confidence level when the data meets the assumptions of normality and homogeneity of variance. Another approach is to consider using the non-parametric rank-sum test when the two sets of data do not meet the normality condition. The chi-square test was used to assess differences in cell cycle percentages. A probability (*p*) value of < 0.05 was considered statistically significant. NS *p* > 0.05, *p < 0.05, **p < 0.01, and ***p < 0.001 indicated different degrees of statistical significance, as noted in the figures.

## Supplementary information

supplementary legends

original western blot

supplemental Figure 1

supplemental Figure 2

supplemental Figure 3

## Data Availability

The datasets used in the current study are available from the corresponding author on reasonable request.
