# Supplementary material for: The dual HDAC/PI3K inhibitor CUDC-907 inhibits the growth and proliferation of MYC-driven Group 3 medulloblastoma
Source: Cell Death Discov. 2025 Apr 14;11:172. doi: 10.1038/s41420-025-02470-4 (PMC11997184; doi:10.1038/s41420-025-02470-4)
Supplement: Supplementary file 1 — supplementary legends [file 41420_2025_2470_MOESM1_ESM.docx]

**Supplementary Fig. 1 A-C** The impact of MYC expression levels on the prognosis of WNT, SHH and G4 MB. **D-I** Pearson correlation analysis between the expression levels of MYC and HDAC/PI3K pathway-related proteins. **J** G3 MB cell (MB230524) was screened out from the patient-derived primary cells. **K** Methylation gene testing report of G3 MB patient with MYC amplification. **L** The MRI of whole brain and spinal cord showed tumor dissemination and metastasis.

**Supplementary Fig. 2 A** The effect of CUDC-907, Entinostat and LY294002 on D283 cells viability was measured and compared. **B** The effect of CUDC-907, Entinostat and LY294002 on MB230524 cells.

**Supplementary Fig. 3 A** Bright field plot and growth curve of MBOs after CUDC-907 gradient processing. ***p < 0.001.
