# Supplementary figures and images for: The dual HDAC/PI3K inhibitor CUDC-907 inhibits the growth and proliferation of MYC-driven Group 3 medulloblastoma
Source: Cell Death Discov. 2025 Apr 14;11:172. doi: 10.1038/s41420-025-02470-4 (PMC11997184; doi:10.1038/s41420-025-02470-4)

Fig 1B


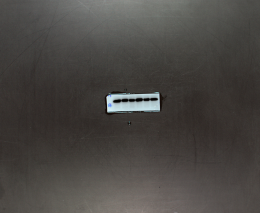

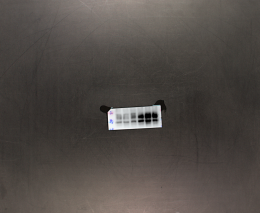

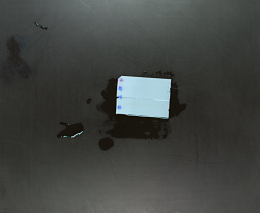


Fig 1D


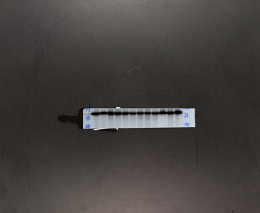

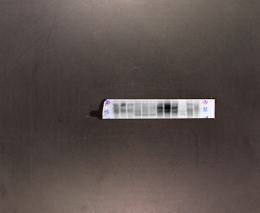

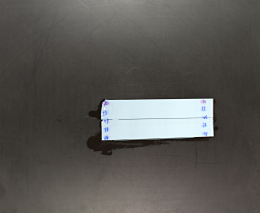


Fig 1L


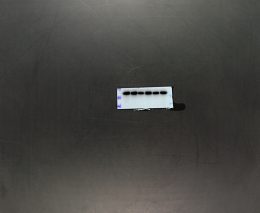

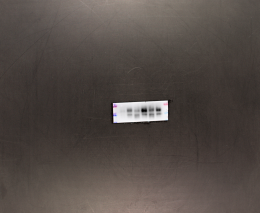

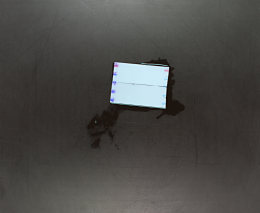


Fig 2E


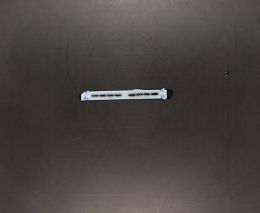

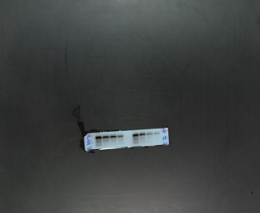

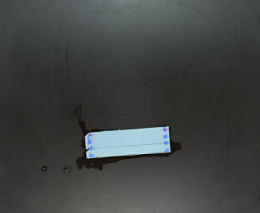


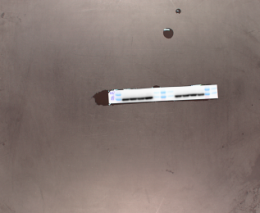

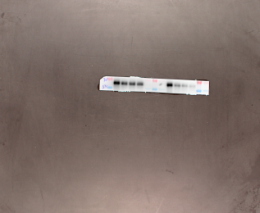

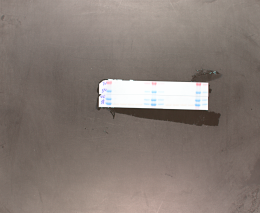


Fig 2F


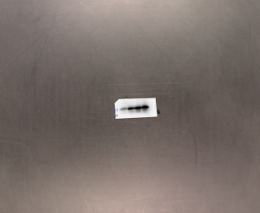

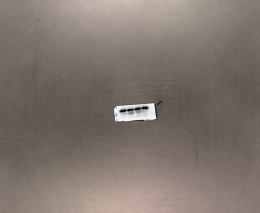

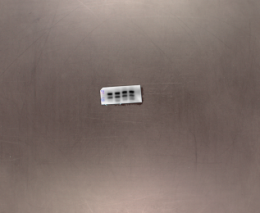

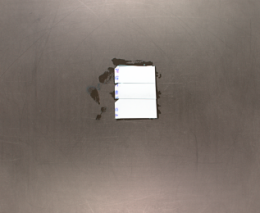


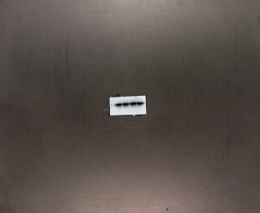

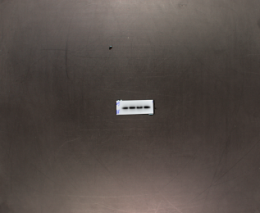

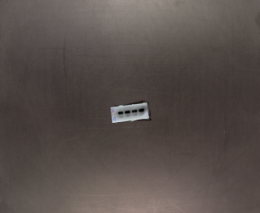

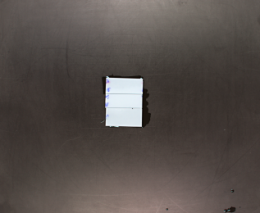


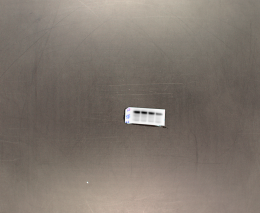

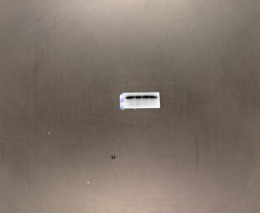

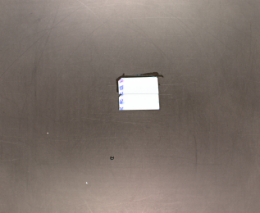


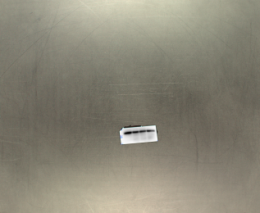

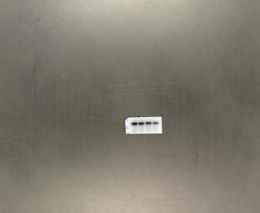

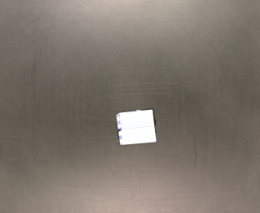


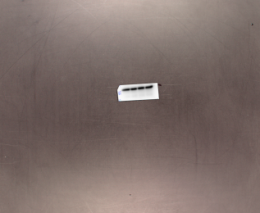

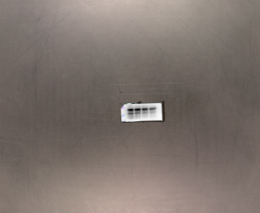

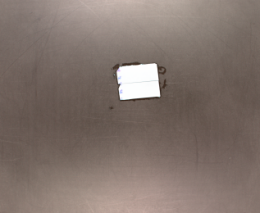


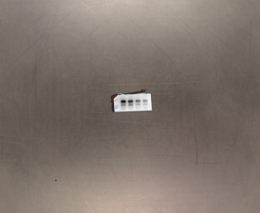

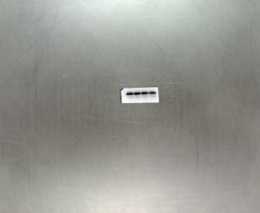

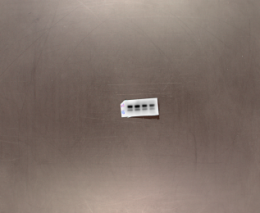

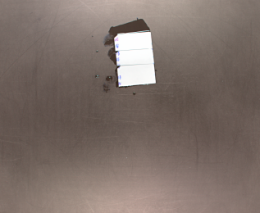


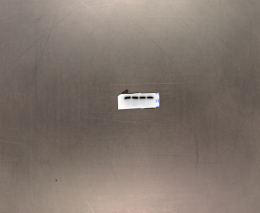

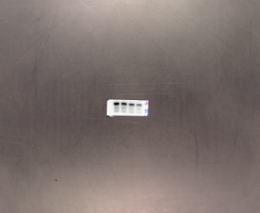

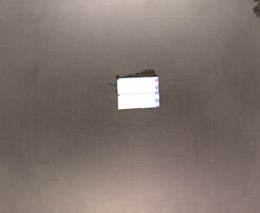


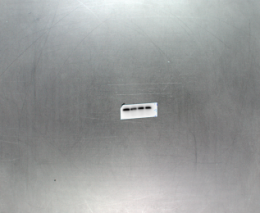

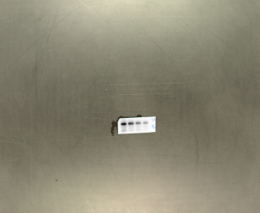

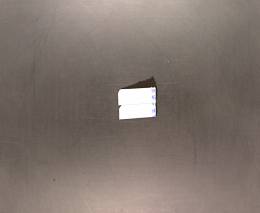


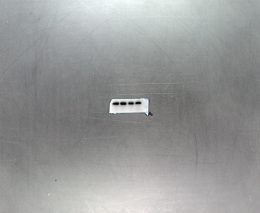

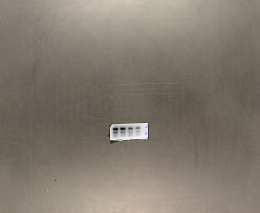

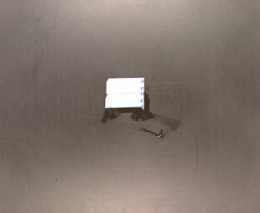


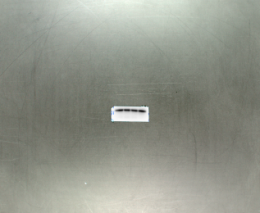

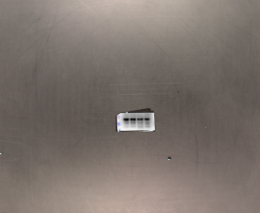

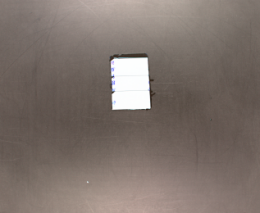


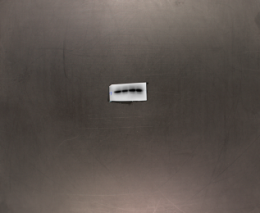

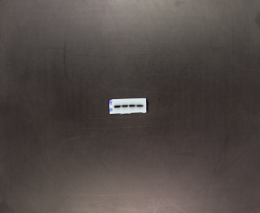

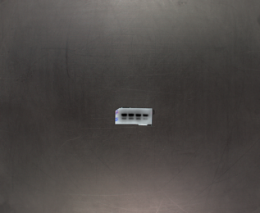

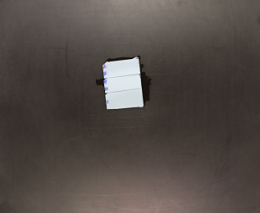


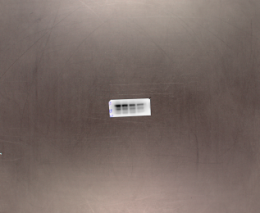

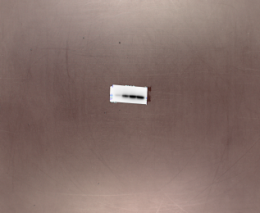

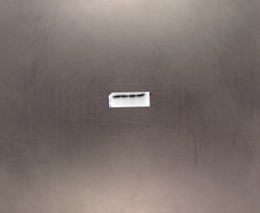

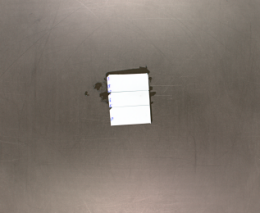


Fig 3C


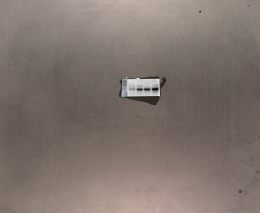

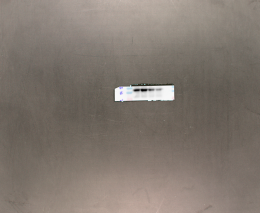

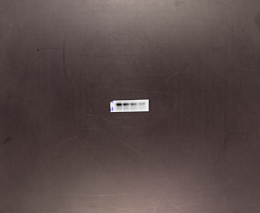

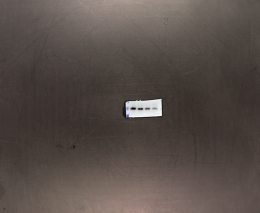

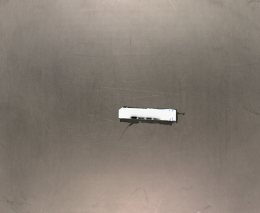

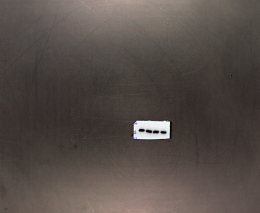

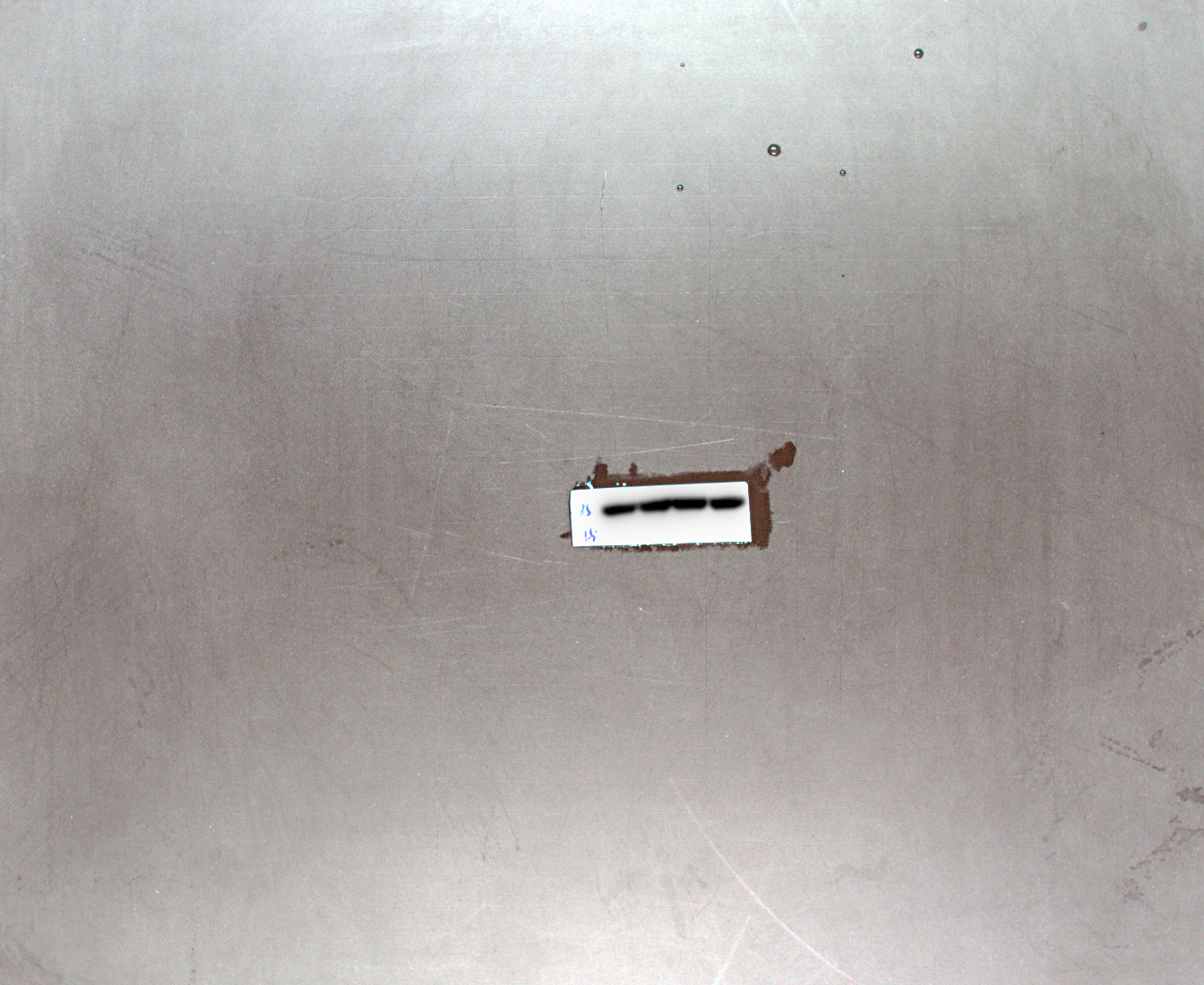

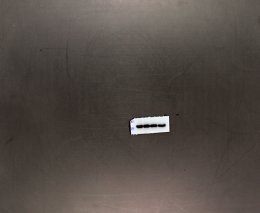

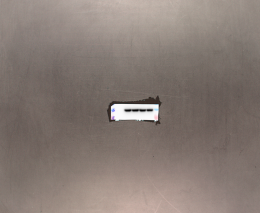

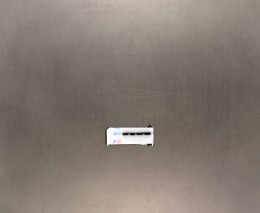

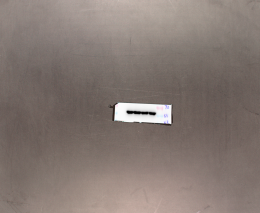

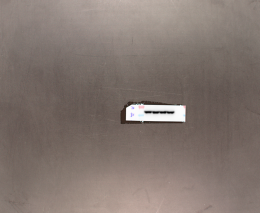

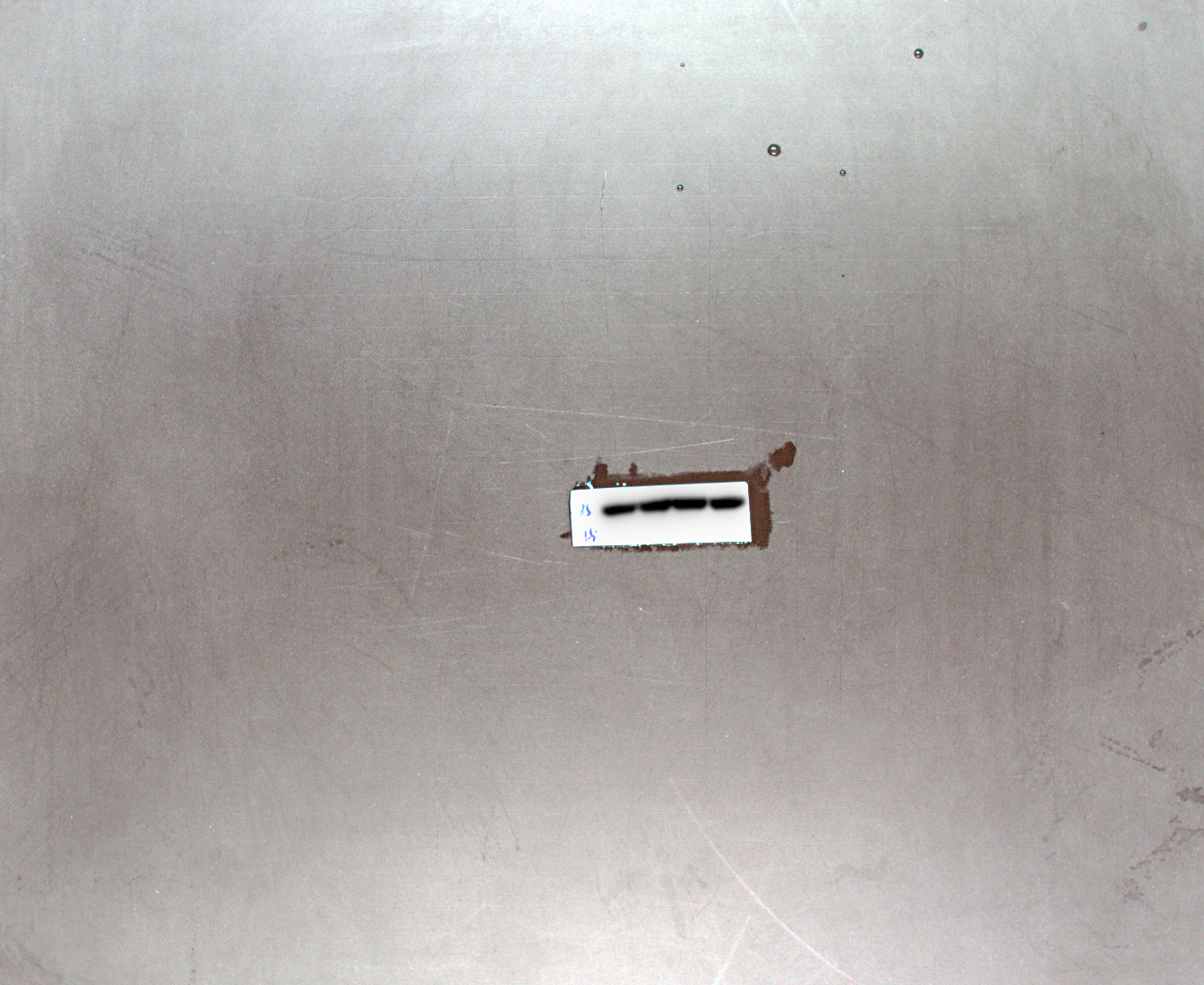

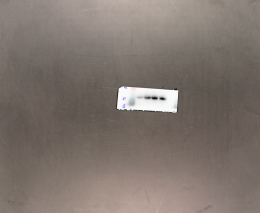

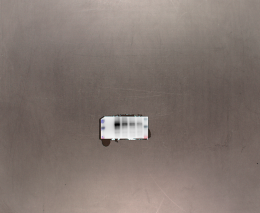

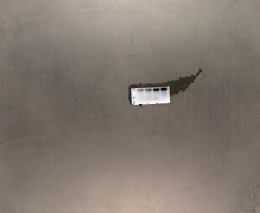

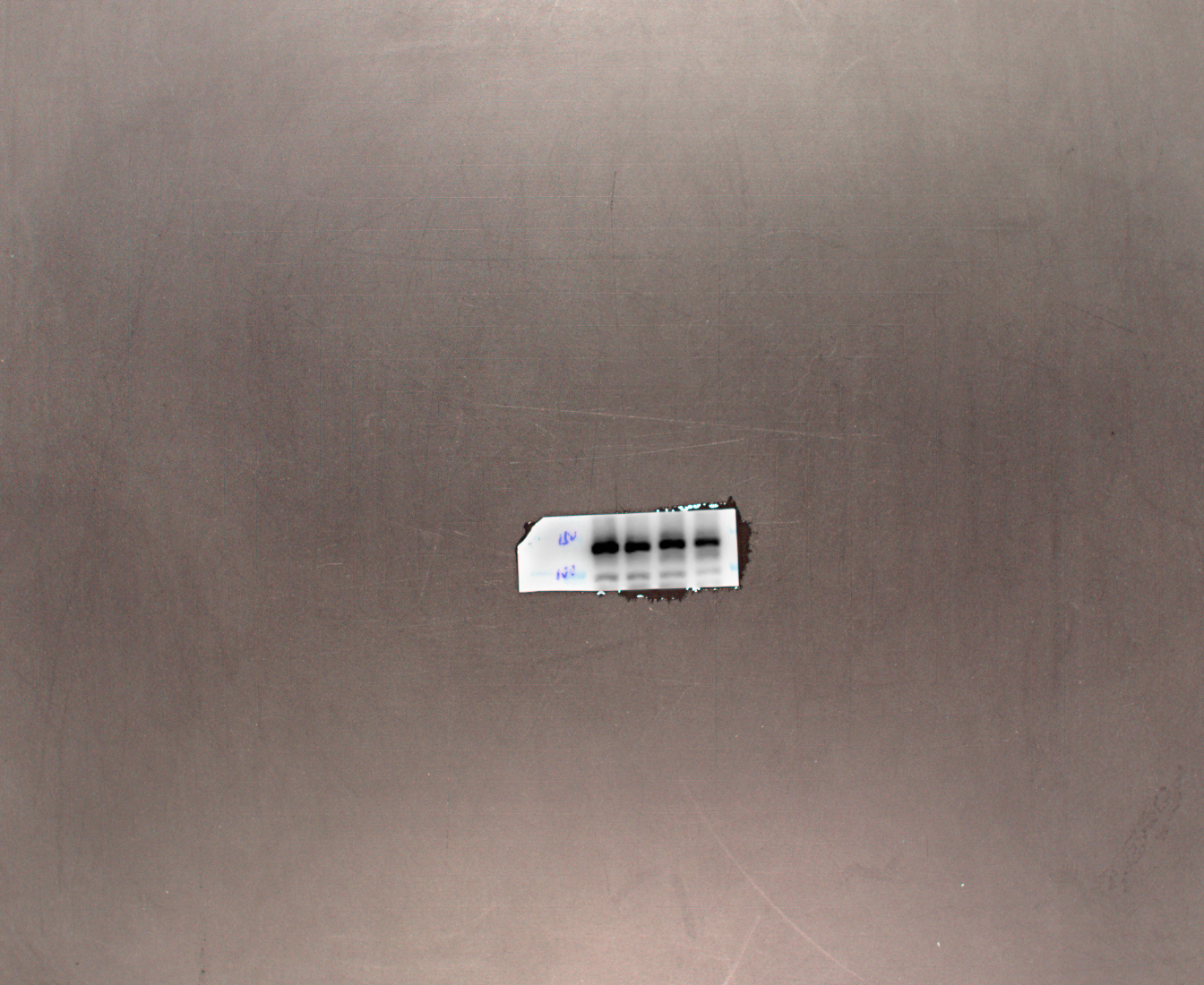

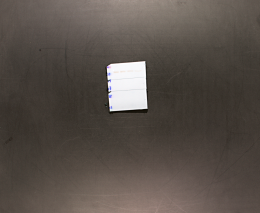

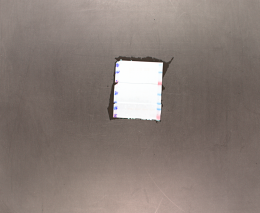

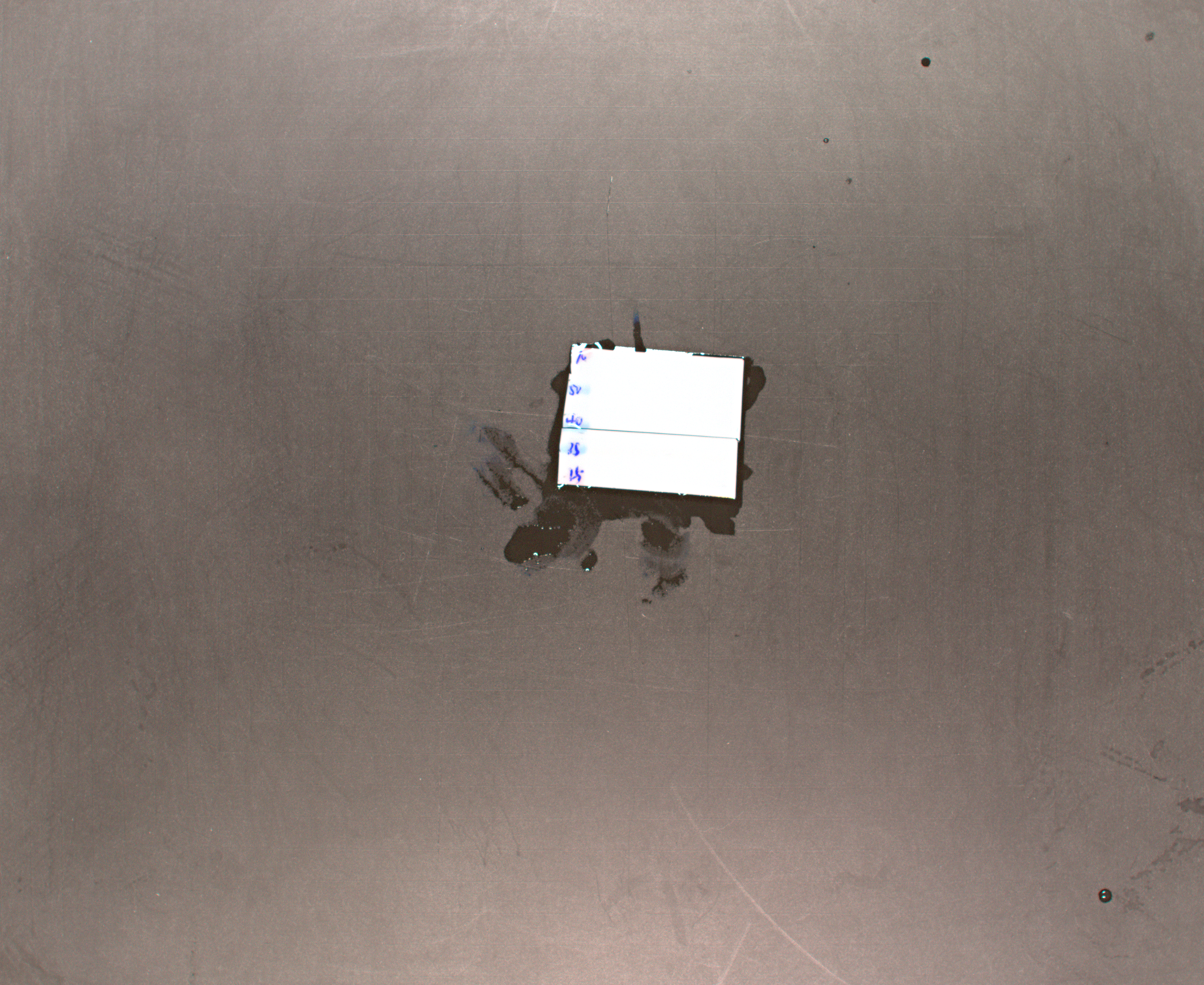

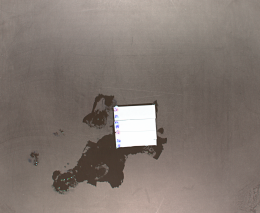

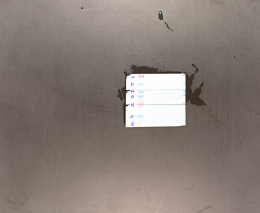

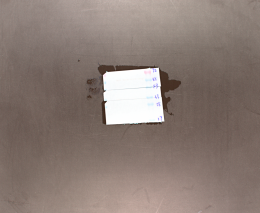

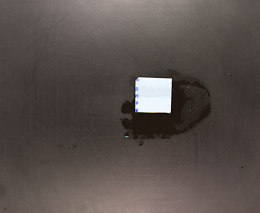


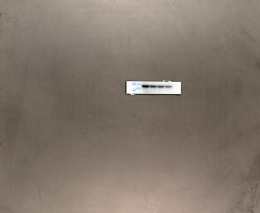

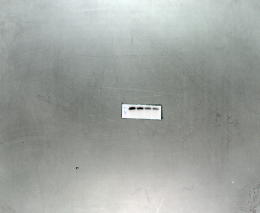

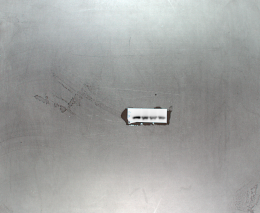

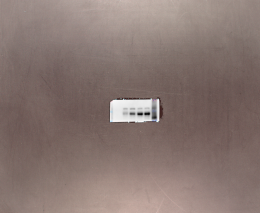

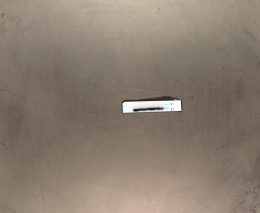

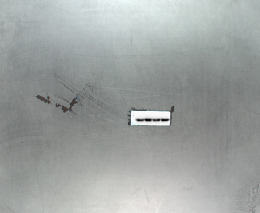

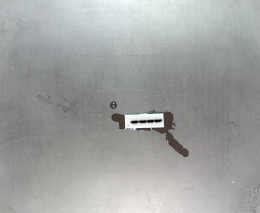

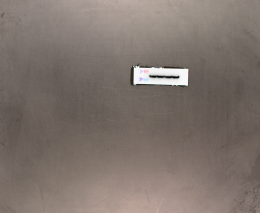

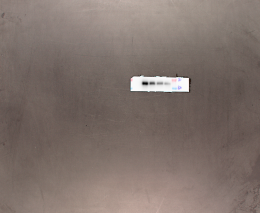

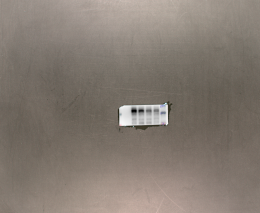

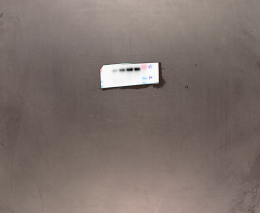

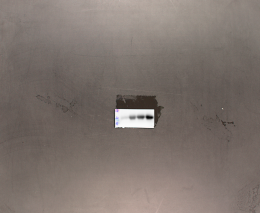

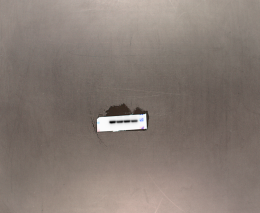

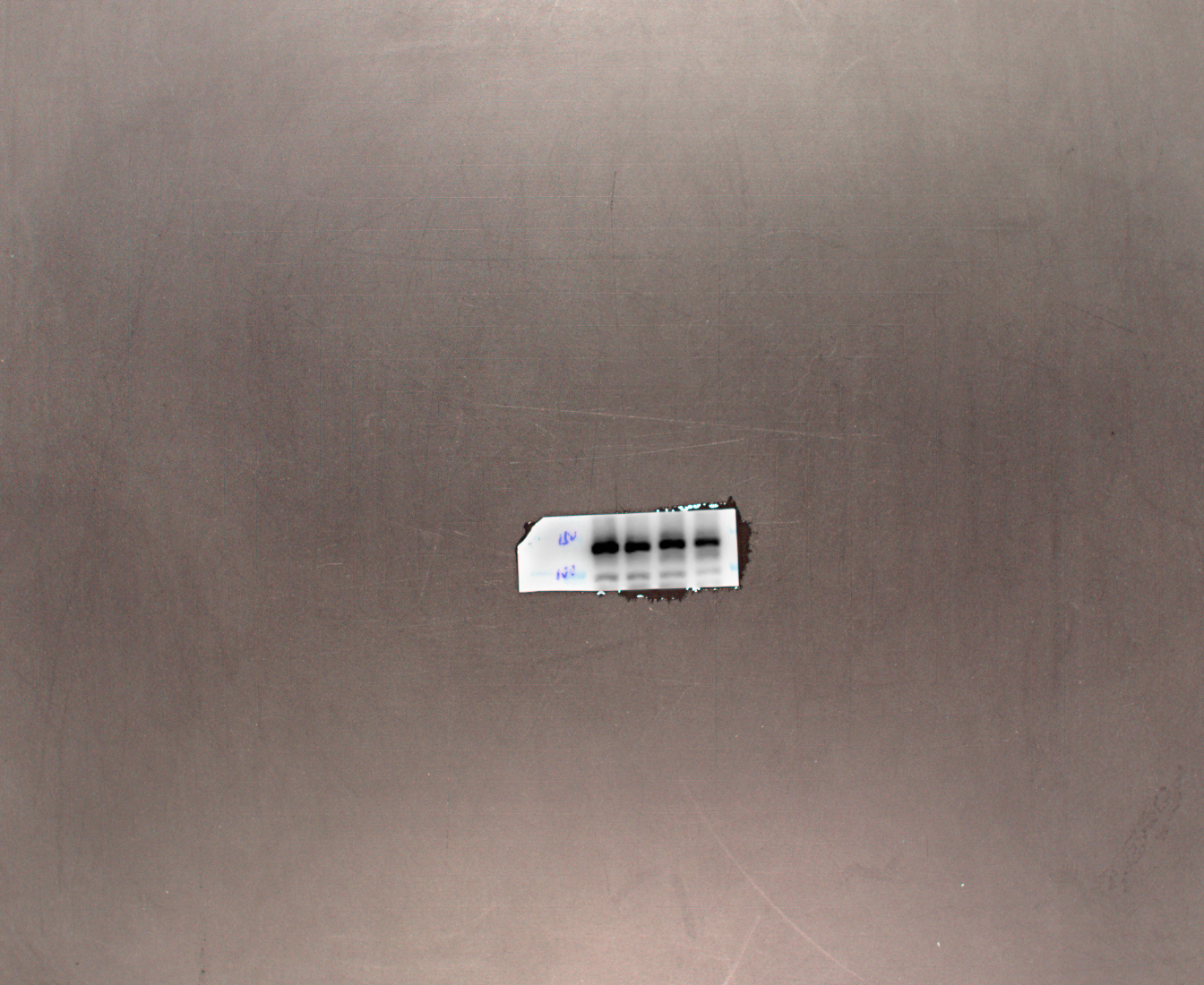

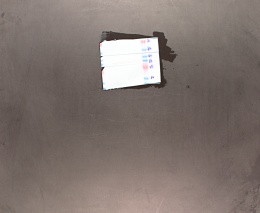

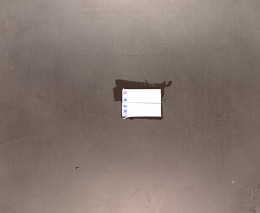

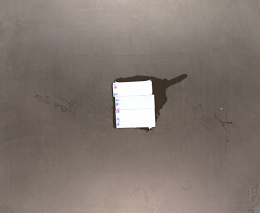

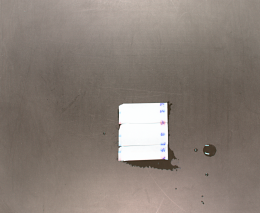

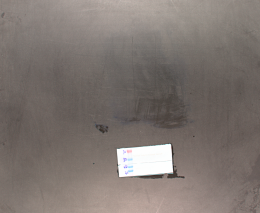


Fig 3D


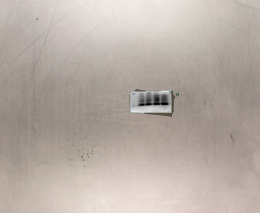


Fig 3F

Fig 3H

Fig 5B

Fig 5C

Supplement: Supplementary file 2 — original western blot [file 41420_2025_2470_MOESM2_ESM.docx]

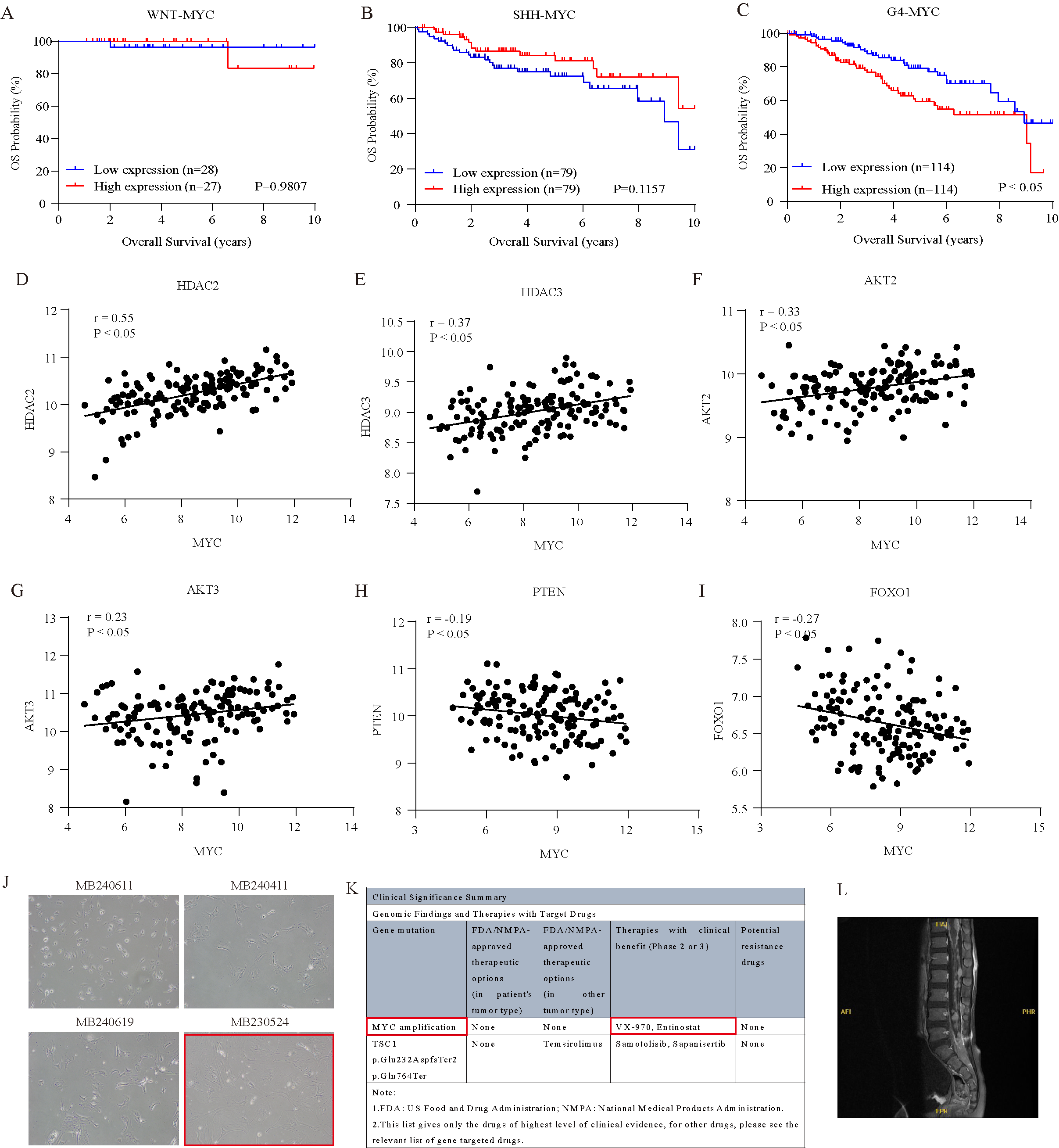

Supplement: Supplementary file 3 — supplemental Figure 1 [file 41420_2025_2470_MOESM3_ESM.tif]

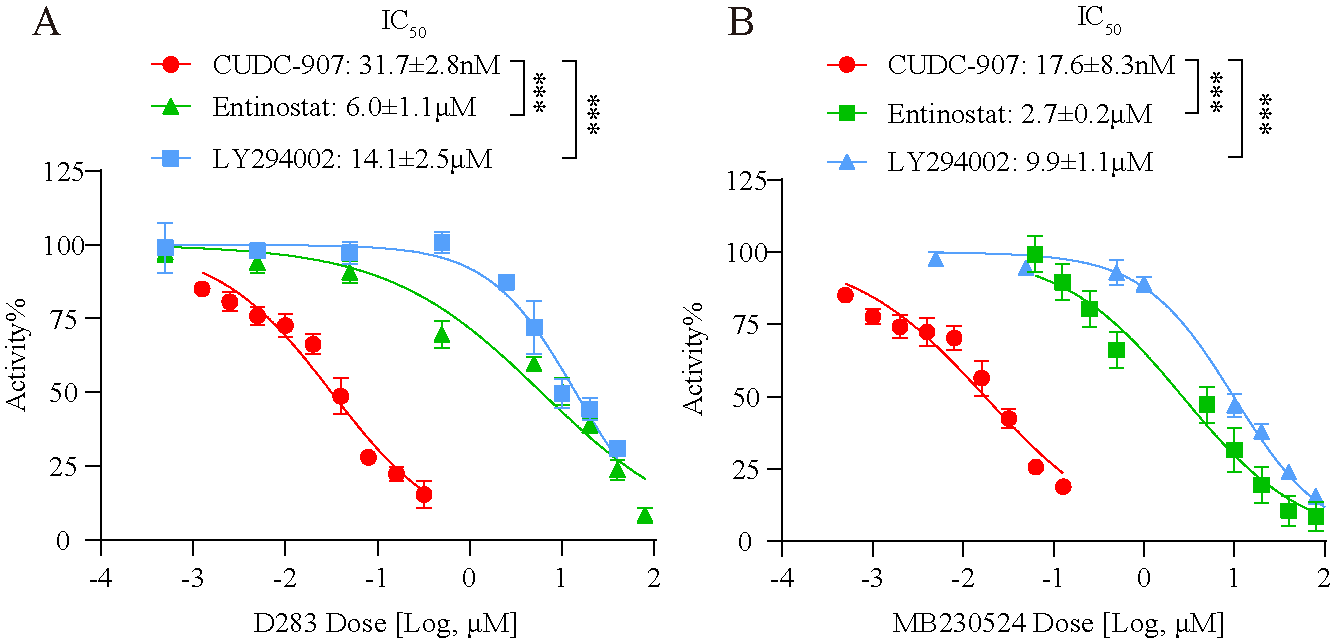

Supplement: Supplementary file 4 — supplemental Figure 2 [file 41420_2025_2470_MOESM4_ESM.tif]

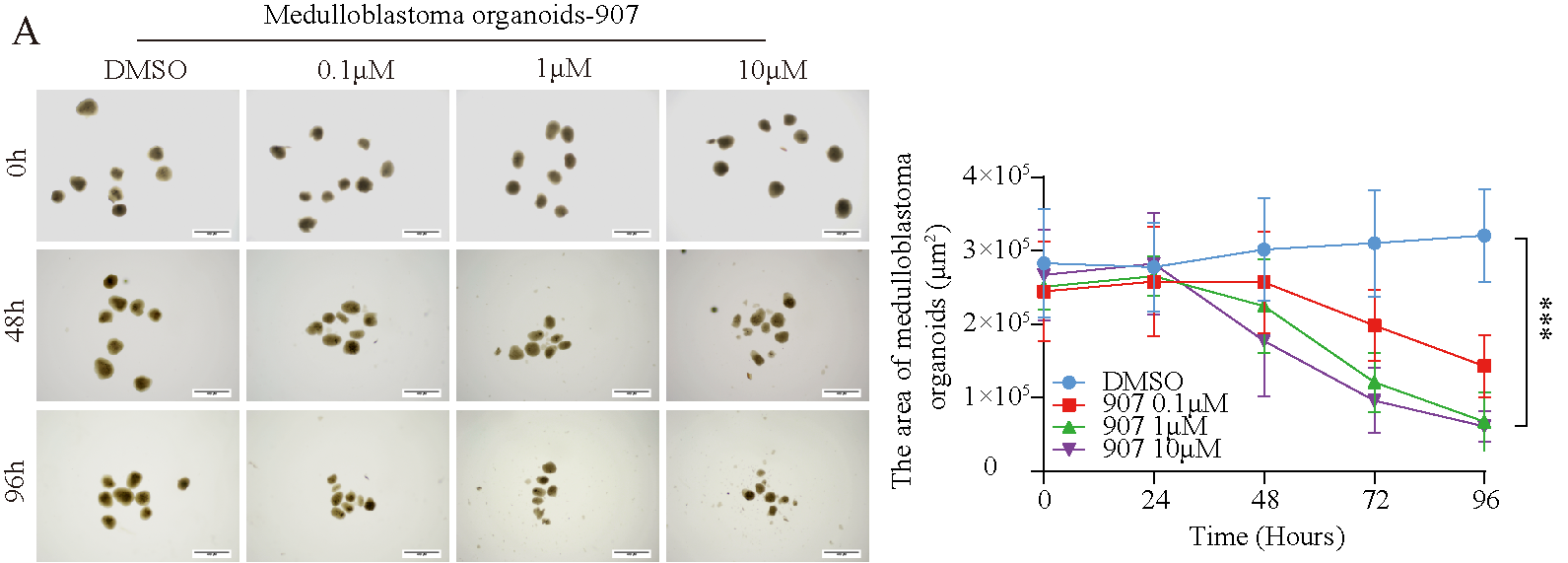

Supplement: Supplementary file 5 — supplemental Figure 3 [file 41420_2025_2470_MOESM5_ESM.tif]
